# Does Respect Foster Tolerance? (Re)analyzing and Synthesizing Data From a Large Research Project Using Meta-Analytic Techniques

**DOI:** 10.1177/01461672211024422

**Published:** 2021-06-19

**Authors:** Steffen Zitzmann, Lukas Loreth, Klaus Michael Reininger, Bernd Simon

**Affiliations:** 1 University of Tübingen, Germany; 2 Kiel University, Germany; 3 University Medical Center Hamburg-Eppendorf, Germany

**Keywords:** disapproval, respect, tolerance, meta-analysis

## Abstract

Our own prior research has demonstrated that respect for disapproved others predicts and might foster tolerance toward them. This means that without giving up their disapproval of others’ way of life, people can tolerate others when they respect them as equals (outgroup respect–tolerance hypothesis). Still, there was considerable variation in the study features. Moreover, the studies are part of a larger research project that affords many additional tests of our hypothesis. To achieve integration along with a more robust understanding of the relation between respect and tolerance, we (re)analyzed all existing data from this project, and we synthesized the results with the help of meta-analytic techniques. The average standardized regression coefficient, which describes the relationship between respect and tolerance, was 0.25 (95% confidence interval [CI] = [0.16, 0.34]). In addition to this overall confirmation of our hypothesis, the size of this coefficient varied with a number of variables. It was larger for numerical majorities than for minorities, smaller for high-status than for low-status groups, and larger for religious than for life-style groups. These findings should inspire further theory development and spur growth in the social-psychological literature on tolerance.

As reflected in many political and societal debates, managing diversity has become increasingly challenging for pluralistic societies. Besides the many positive aspects that come with diversity (e.g., cultural exchange and learning), there is also the risk that, driven by categorizations of others into “us” or “them,” diversity can lead to conflicts between groups (intergroup conflict). This has led to calls for mutual tolerance (Scanlon, 2003). However, many scholars (e.g., Simon, Eschert, et al., 2019; Verkuyten & Yogeeswaran, 2017; Vogt, 1997) have pointed out that tolerance has scarcely been addressed systematically in social-psychological theorizing and empirical research.

In social psychology, tolerating others has long been thought to involve liking their beliefs, preferences, and practices, or regarding them as something good (see Simon, Eschert, et al., 2019). However, in recent years, social psychologists have adopted ideas from philosophy (e.g., from Scanlon, 2003; see also Forst, 2013) that have gradually shifted their understanding toward a theoretically more sophisticated view of tolerance as the attitude that one accepts the different ways of life practiced by outgroups (i.e., their beliefs, preferences, and practices) despite one’s disapproval of them. According to this emerging view, disapproval of others (i.e., of their ways of life) is a definitional condition for tolerance. In other words, it makes sense to say that one tolerates other people or things only when one first disapproves of them (e.g., Gibson et al., 1992). Whereas tolerance is a popular and loaded word not only in everyday social and political life but also in social scientific discourses, a great deal of controversy surrounds the exact meaning of tolerance. This situation testifies to the “difficulty of tolerance” (Scanlon, 2003) as an interpretive concept, which allows for rival interpretations (Dworkin, 2013). In other words, tolerance is a contested concept, which allows and in fact calls for competing conceptions. Note that we do not aspire to dictate or prescribe a specific definition of tolerance. Rather, we offer a particular conception of tolerance that sheds new light on the processes and phenomena associated with tolerance and thus helps to deepen our understanding of the value of tolerance but also of the controversies that surround it.

On the basis of this understanding of tolerance, social psychologists have begun to study the sources behind tolerance. One source is associated with the multilevel nature of social categorization processes. Whereas groups often differ in their ways of life, they share membership in higher level groups such as a common society or nation-state (Turner et al., 1987). Such a common higher level group membership then operates as a social-psychological source of a mutual recognition of equality (and corresponding entitlements) across lower level group boundaries (Simon, 2020). Members of different (lower level) groups can thus recognize each other as equals at the higher level (e.g., as fellow citizens with the same rights, duties, and liberties).

Moreover, in line with Honneth’s (1995) recognition theory, which assumes that equality recognition underlies respect, Simon and Grabow (2014) found that out of the three recognition principles (need, equality, and achievement recognition), equality recognition was indeed most predictive of the experience of being respected (see also Simon et al., 2015). Therefore, respect is hereafter also referred to as respect for others as equals or equality-based respect (see, for example, Eschert & Simon, 2019; Renger et al., 2017). Notice that unlike alternative conceptions (e.g., Huo & Molina, 2006), according to the definition employed by us here, respecting others as equals is not at odds with disapproving of them (see Crane, 2017; Scanlon, 2003; see also Simon, Eschert, et al., 2019; Verkuyten et al., 2019); that is, one can respect others as equal citizens, while disapproving of their ways of life.

The role of respect in tolerance has been highlighted by the disapproval-respect model of tolerance (DRM; Simon & Schaefer, 2016). The central hypothesis derived from this model is the outgroup respect–tolerance hypothesis. According to this hypothesis, people’s respect for outgroups they disapprove of influences their tolerance toward these groups. In line with the hypothesis, Simon and Schaefer (2016) found that the respect paid by Muslims in Germany to groups they disapprove of predicted the Muslims’ tolerance toward these groups. More recently, using a longitudinal research design with time-lagged measures of tolerance, Simon, Eschert, et al. (2019) showed that Tea Party supporters’ respect for groups they disapprove of can foster the development of tolerance toward these groups over time (Study 1). Moreover, experimental data have suggested that respect even causally influences tolerance (Simon, Eschert, et al., 2019; Study 2).

However, although empirical research has supported the notion that tolerance and respect are related to one another, the sizes reported for this relation (i.e., the standardized regression coefficients) have varied considerably, possibly due to differences between studies, which exist, for example, in the sample or the design of the study. Therefore, the aim of the present study was to disentangle these sources of variation and, thus, to provide a more comprehensive picture of the relation between respect and tolerance. To this end, we (re)analyzed the data that were collected for a large multistudy research project directed by the last author and synthesized the results with the help of meta-analytic techniques while taking study variability into account. We investigated the following substantive research questions and methodological questions. Because our overall aim was to test the outgroup respect–tolerance hypothesis, we first asked whether respect would predict tolerance on average (aggregated across the results for this relation).

Second, we also examined how the results would vary in accordance with the features of the studies or the data sets. Exploring moderators considered in theoretical models is one way to enrich theoretical reasoning and to contribute to the further development of these models.

Therefore, we investigated moderators of theoretical importance, which is novel to the research on the DRM but quite common in social-psychological research in general. For example, Tropp and Pettigrew (2005) found that the relationship between the variables that they studied differed between members of a majority group and members of a minority group, which the authors explained by a psychological asymmetry between these two groups. The DRM in its current form does not predict that—or how—the respect–tolerance link should vary as a function of people’s social position in society at large, although such variables may affect the level of tolerance expressed toward outgroups (Simon et al., 2001). Nevertheless, given the role of these variables in other social-psychological models and research, one may also speculate about their moderating influence on the respect–tolerance link. It stands to reason that, for example, the members of a numerical majority or a high-status group, which are particularly likely to regard themselves as prototypical of and thus normative for society at large, may be in greater need of a good reason to tolerate the “deviant” others. Respect for them should be such a reason so that the more respect members of majorities or high-status groups actually grant them, the stronger their reason for tolerance toward them. In other words, we speculated that tolerance among members of majorities or high-status groups could be more dependent on explicit reasoning or considerations concerning their respect for others which should then be reflected in a stronger respect–tolerance link relative to that for members of minority or low-status groups. The same could be true for religious groups. Relative to non-religious groups, they may also be in greater need of a good reason to tolerate those who fail to see “the Godly truths.” Taken together, these group membership variables lend themselves as plausible candidates to an empirical investigation of potential moderator variables.

Moreover, we were interested in whether the methodology employed in the different studies would affect the respect–tolerance link. To give an example, to test the outgroup respect–tolerance hypothesis, Simon and Schaefer (2016) employed a cross-sectional design, which—as with all correlational studies—relied on the naturally occurring variation between variables. Unlike this study, Simon, Eschert, et al. (2019) employed a longitudinal design (Study 1). However, because longitudinal studies are still vulnerable to unwanted confounding by unobserved third variables, the authors additionally conducted an experiment (Study 2) that allowed for more control by means of randomization. Thus, we asked whether the design would explain variation in results for the respect–tolerance link.

In statistical terms, one way to model this link is with a linear model with tolerance as the dependent variable and a measure of respect or a manipulation thereof as the predictor variable in cross-sectional or experimental designs and, in longitudinal designs, often also with an additional measure of tolerance as the lagged criterion. At the same time, one might wish to exclude reasonable alternative explanations by adding one or more “third variables” as covariates to the model. Even in the analysis of experimental data, it might be helpful to include such covariates, particularly when randomization is not perfect and confounding cannot be precluded. We thus asked whether choosing a model with covariates would affect the results.

Finally, constructs in social psychology cannot be measured without error. A person’s attitude or belief could be assessed by asking him or her to rate the respective variable either on a single item or across multiple items, which are then aggregated into a scale score. From a measurement perspective, the scale score can be more reliable than a single item. However, in general, neither the single item nor the scale score can provide a perfectly reliable measure, and if errors in the predictor variables are ignored, regression coefficients will be biased (e.g., Fuller, 1987). To mitigate this problem, one can adopt a latent-variable approach, which models a variable’s true score and thus accounts for the measurement error. In fact, the research considered in this article has used different methods to assess respect. Therefore, in addition to the analysis models with or without covariates, our analytic approach covers three types of measures. We raised the question of whether these types would yield results of varying size.^1^ Specifically, we raised the question of whether the use of the latent variable would yield larger sizes of the relation between respect and tolerance than the use of the scale score or a single item, and whether the use of the scale score would yield larger sizes than the use of a single item. Note that these are directed hypotheses.

## The Present Work

Recent social-psychological research has indicated that respect for others of whom one disapproves influences one’s tolerance toward them. The aim of the present article is twofold: First, despite a slow start, empirical work on the respect–tolerance link has been accumulating, and several studies have been published in the past few years (e.g., Simon, Eschert, et al., 2019; Simon & Schaefer, 2016). The present work was aimed at summarizing this emerging trend. Specifically, we tested whether, on average, respect influences tolerance, and we disentangled sources of variation in the strength of the respect–tolerance link.

The added value of our study is noteworthy. Employing meta-analytic techniques, we synthesized a comprehensive corpus of research on the relation between respect and tolerance, and we believe this is an important milestone for future research on tolerance in general. Moreover, we conducted several moderator analyses, some of which were theoretically motivated, whereas others were more exploratory in nature. We believe that studying moderators and finding evidence for moderating effects can inform future study designs in important ways, contribute to the development of interventions for promoting tolerance, and promote scientifically informed debates in society at large on the important topic of mutual tolerance.

In line with the DRM, we expected that the average size of the respect–tolerance link would be positive and substantial (main research question). Theoretical and methodological thinking (see above) led us to investigate a number of possible substantive and methodological moderators, which are summarized in Table 1 (see also the “Coding” section). For exploratory reasons, we also included a number of other potential moderators. These analyses were exploratory because theoretical assumptions about how these variables would influence the relation between respect and tolerance did not exist. Therefore, we did not state more specific research questions for these variables.

**Table 1.** Overview of Moderators.

Type of moderator	Moderator	Categories
Substantive	Numerical minority or majority (sample)	Minority
		Majority
	Numerical minority or majority (outgroup)	Minority
		Majority
	Social status (sample)	Low
		High
	Social status (outgroup)	Low
		High
	Type of group (sample)	Political
		Religious
		Life-style
	Type of group (outgroup)	Political
		Religious
		Life-style
		Ethnic
Methodological	Design	Cross-sectional
		Longitudinal
		Experimental
	Analysis model	No covariates
		Covariates included
	Respect measure	Single item
		Scale score
		Latent variable
Additional	Country of residence^a^	Germany
		Poland
		The United States
		Brazil
	Sample^a^	Tea Party supporters
		Catholics
		Protestants
		Muslims
		Alevis
		LGBTs
		University students
	Mean age^a^	—
	Percentage of women^a^	—
	Number of participants^a^	—

*Note.* LGBTs = lesbian, gay, bisexual, and transgender individuals.

a Moderators included for exploratory reasons.

## Method

### Data Retrieval and Preprocessing

The data came from a large research project directed by the last author concerning life in pluralistic societies. To gain access to the data from this project, we asked contributors to the research project to submit the data sets from their already published (Krys et al., 2020; Paffrath & Simon, 2020; Schaefer & Simon, 2019; Simon, Eschert, et al., 2019; Simon et al., 2016, 2019; Simon & Schaefer, 2016, 2018) or not yet published articles (Schaefer et al., 2021) that included the relevant measurements for integration into our analysis. Besides measures of tolerance and approval, the data sets had to contain at least one measure of respect. We received a total of 11 distinct, large data sets in response to the request, and we split these into smaller subsets. An overview is provided in Table 2, which shows the relevant features of the requested data (sub)sets. For more details, see the Supplemental Material.

**Table 2.** Selected Features of the Requested Data Sets.

Study ID	Design	Sample characteristics				Outgroup	Standardized regression coefficient			
		Sample	Country of residence	Mean age	Percentage of women		Est.	*SE*s	L95%CI	U95%CI
#01 (data used by Schaefer et al., 2021; Simon & Schaefer, 2016)	Cross-sectional (T1)	Muslims	Germany	30.39	49.1	Atheists	0.57	0.03	0.51	0.64
				31.17	35.8	Christians	0.61	0.07	0.47	0.76
				29.97	40.6	Feminists	0.58	0.04	0.50	0.66
				29.91	42.8	Homosexuals	0.50	0.04	0.42	0.57
				30.30	38.0	Jews	0.69	0.05	0.58	0.79
	Cross-sectional (T2)	Muslims	Germany	30.92	48.9	Atheists	0.65	0.02	0.61	0.70
				30.85	48.2	Christians	0.28	0.03	0.22	0.34
				30.87	47.7	Feminists	0.39	0.03	0.33	0.45
				30.80	48.1	Homosexuals	0.58	0.02	0.53	0.62
				30.81	48.5	Jews	0.71	0.02	0.67	0.76
	Longitudinal (T1–T2)	Muslims	Germany	30.47	49.6	Atheists	0.37	0.04	0.28	0.46
				31.24	33.1	Christians	0.18	0.12	−0.06	0.43
				30.08	39.6	Feminists	0.60	0.05	0.51	0.69
				29.93	42.8	Homosexuals	0.12	0.04	0.04	0.21
				30.50	36.4	Jews	−0.05	0.11	−0.27	0.16
#02 (data used by Krys et al., 2020; Schaefer et al., 2021; Simon et al., 2016)	Cross-sectional (T1)	Protestants	Brazil	40.36	31.8	Afro-Brazilians	0.30	0.07	0.16	0.43
				40.20	30.4	Atheists	0.36	0.05	0.26	0.47
				41.49	28.6	Catholics	0.29	0.10	0.10	0.48
				39.95	29.9	Feminists	0.17	0.06	0.05	0.29
				40.03	30.1	Homosexuals	0.25	0.06	0.13	0.36
	Cross-sectional (T2)	Protestants	Brazil	39.63	28.6	Afro-Brazilians	0.30	0.05	0.20	0.40
				39.64	29.2	Atheists	0.30	0.05	0.19	0.40
				39.65	28.2	Catholics	0.10	0.05	−0.01	0.21
				39.78	28.9	Homosexuals	−0.07	0.06	−0.19	0.04
	Longitudinal (T1–T2)	Protestants	Brazil	40.36	31.8	Afro-Brazilians	0.06	0.05	−0.04	0.16
				40.20	30.4	Atheists	0.07	0.05	−0.02	0.17
				41.49	28.6	Catholics	0.66	0.04	0.59	0.73
				40.03	30.1	Homosexuals	0.01	0.05	−0.07	0.10
#03 (data used by Krys et al., 2020; Schaefer et al., 2021)	Cross-sectional (T1)	Protestants	Germany	49.55	46.6	Atheists	0.28	0.03	0.21	0.35
				47.40	48.7	Catholics	0.31	0.07	0.18	0.44
				48.55	42.4	Homosexuals	0.32	0.04	0.24	0.39
				48.55	42.4	Muslims	0.27	0.04	0.20	0.34
	Cross-sectional (T2)	Protestants	Germany	50.55	47.5	Atheists	0.37	0.03	0.32	0.43
				50.71	47.3	Catholics	0.36	0.03	0.30	0.42
				50.56	46.3	Homosexuals	0.36	0.03	0.30	0.42
				50.61	46.6	Muslims	0.36	0.03	0.30	0.42
	Longitudinal (T1–T2)	Protestants	Germany	49.14	46.4	Atheists	0.14	0.03	0.07	0.20
				46.76	46.2	Catholics	0.26	0.06	0.13	0.38
				48.36	42.1	Homosexuals	0.13	0.04	0.05	0.21
				48.29	42.1	Muslims	0.05	0.04	−0.03	0.13
#04 (data used by Reininger et al., 2020; Schaefer et al., 2021; Simon, Eschert, et al., 2019; Simon, Reininger, et al., 2019)	Cross-sectional (T1)	Tea Party supporters	The United States	44.51	46.0	Conservatives	0.65	0.11	0.43	0.87
				53.17	40.9	Homosexuals	0.50	0.04	0.42	0.58
				54.31	40.1	Liberals	0.42	0.04	0.35	0.49
				53.40	43.7	Muslims	0.55	0.03	0.49	0.61
	Cross-sectional (T2)	Tea Party supporters	The United States	49.44	49.6	Conservatives	0.13	0.04	0.05	0.21
				51.24	47.2	Homosexuals	0.51	0.03	0.45	0.57
				52.33	44.9	Liberals	0.50	0.03	0.44	0.57
				51.69	46.6	Muslims	0.59	0.03	0.53	0.65
	Cross-sectional (T3)	Tea Party supporters	The United States	50.53	47.2	Conservatives	0.06	0.04	−0.01	0.14
				51.44	46.0	Homosexuals	0.53	0.03	0.47	0.58
				52.14	45.0	Liberals	0.45	0.03	0.39	0.50
				51.84	45.8	Muslims	0.54	0.03	0.48	0.60
	Longitudinal (T1–T2)	Tea Party supporters	The United States	52.81	42.0	Homosexuals	0.22	0.04	0.14	0.31
				54.47	40.5	Liberals	0.30	0.04	0.23	0.37
				53.18	44.6	Muslims	0.22	0.04	0.14	0.30
	Longitudinal (T1–T3)	Tea Party supporters	The United States	53.09	41.1	Homosexuals	0.36	0.04	0.28	0.44
				54.41	39.9	Liberals	0.25	0.04	0.18	0.33
				53.40	43.9	Muslims	0.18	0.04	0.10	0.27
	Longitudinal (T2–T3)	Tea Party supporters	The United States	51.03	47.2	Homosexuals	0.28	0.04	0.21	0.34
				52.30	45.2	Liberals	0.17	0.03	0.11	0.24
				51.63	46.4	Muslims	0.14	0.04	0.07	0.22
#05 (data used by Schaefer et al., 2021)	Cross-sectional (T1)	Alevis	Germany	28.54	52.7	Atheists	0.45	0.08	0.29	0.62
				29.81	35.4	Feminists	0.60	0.09	0.42	0.78
				32.13	30.8	Homosexuals	0.58	0.08	0.43	0.73
				31.93	46.4	Jews	0.63	0.09	0.45	0.82
				32.51	42.9	Sunnis	0.41	0.07	0.28	0.54
	Cross-sectional (T2)	Alevis	Germany	30.60	49.9	Atheists	−0.07	0.05	−0.17	0.03
				30.61	49.0	Christians	0.00	0.05	−0.10	0.09
				30.55	49.2	Feminists	0.04	0.05	−0.05	0.14
				30.66	49.4	Jews	−0.03	0.05	−0.12	0.07
				30.47	48.9	Sunnis	0.19	0.05	0.09	0.28
	Longitudinal (T1–T2)	Alevis	Germany	28.68	51.9	Atheists	0.23	0.08	0.08	0.38
				32.90	41.6	Sunnis	0.29	0.06	0.17	0.40
#06 (data used by Paffrath & Simon, 2020; Schaefer et al., 2021)	Cross-sectional (T1)	Catholics	Poland	40.81	48.1	Atheists	0.55	0.04	0.46	0.63
				39.20	36.7	Feminists	0.52	0.05	0.44	0.61
				40.75	36.2	Homosexuals	0.58	0.04	0.50	0.66
				39.25	48.0	Muslims	0.50	0.04	0.41	0.58
				38.86	43.6	Russian Orthodox people	0.73	0.07	0.60	0.86
	Cross-sectional (T2)	Catholics	Poland	40.33	50.7	Atheists	0.54	0.03	0.48	0.60
				39.97	48.3	Feminists	0.58	0.03	0.52	0.64
				40.11	49.3	Homosexuals	0.70	0.03	0.64	0.75
				40.28	50.8	Muslims	0.40	0.03	0.34	0.46
				39.30	51.6	Russian Orthodox people	0.64	0.03	0.58	0.70
	Longitudinal (T1–T2)	Catholics	Poland	40.33	46.1	Atheists	0.14	0.05	0.03	0.24
				38.38	34.8	Feminists	0.19	0.06	0.08	0.30
				40.42	36.3	Homosexuals	0.28	0.06	0.16	0.39
				38.75	47.5	Muslims	0.16	0.05	0.06	0.26
				37.23	48.9	Russian Orthodox people	−0.18	0.08	−0.34	−0.02
#07 (data used by Schaefer et al., 2021)	Cross-sectional (T1)	LGBTs	The United States	38.97	42.3	African Americans	0.58	0.10	0.38	0.78
				35.00	56.1	Asian Americans	0.45	0.12	0.21	0.68
				38.02	51.2	Latinos/Hispanics	0.57	0.11	0.35	0.80
				33.11	61.0	Tea Party members	0.44	0.04	0.35	0.52
				32.71	59.1	Religious people	0.45	0.06	0.34	0.56
	Cross-sectional (T2)	LGBTs	The United States	33.33	60.7	African Americans	0.15	0.05	0.06	0.24
				33.68	60.6	Latinos/Hispanics	0.13	0.04	0.05	0.22
				33.05	59.9	Tea Party members	0.62	0.04	0.55	0.70
				32.90	59.7	Religious people	0.39	0.04	0.30	0.47
	Longitudinal (T1–T2)	LGBTs	The United States	39.02	38.1	African Americans	−0.53	0.10	−0.72	−0.34
				32.99	61.1	Tea Party members	0.32	0.04	0.23	0.41
				32.55	58.0	Religious people	0.23	0.05	0.12	0.33
#08 (data used by Reininger et al., 2020; Schaefer et al., 2021; Simon, Reininger, et al., 2019)	Cross-sectional (T1)	LGBTs	Germany	40.73	47.5	AfD	0.41	0.03	0.34	0.47
				39.62	44.3	Catholics	0.35	0.05	0.26	0.45
				41.16	21.0	Feminists	0.17	0.13	−0.08	0.41
				40.77	39.3	Muslims	0.42	0.05	0.32	0.52
				39.87	41.0	Protestants	0.34	0.07	0.21	0.48
				39.87	42.4	Refugees	0.39	0.12	0.15	0.62
	Cross-sectional (T2)	LGBTs	Germany	40.56	47.4	AfD	0.45	0.03	0.39	0.51
				39.98	48.3	Catholics	0.20	0.04	0.11	0.29
				40.28	47.8	Feminists	0.74	0.03	0.68	0.81
				40.33	47.1	Muslims	0.36	0.04	0.29	0.44
				39.26	47.2	Protestants	0.12	0.05	0.03	0.21
				40.20	49.0	Refugees	0.19	0.04	0.12	0.27
	Longitudinal (T1–T2)	LGBTs	Germany	40.72	47.7	AfD	0.24	0.03	0.17	0.31
				39.41	43.7	Catholics	−0.11	0.05	−0.21	−0.02
				41.14	21.8	Feminists	−0.09	0.18	−0.44	0.26
				40.70	38.4	Muslims	0.08	0.06	−0.03	0.19
				39.82	40.4	Protestants	−0.04	0.06	−0.16	0.09
				39.97	42.5	Refugees	−2.15	0.23	−2.60	−1.69
#09 (unpublished data)	Cross-sectional (T1)	Catholics	Poland	28.86	65.6	Atheists	0.51	0.05	0.42	0.60
				27.47	57.4	Feminists	0.43	0.05	0.33	0.54
				29.29	68.5	Homosexuals	0.46	0.05	0.37	0.56
				28.45	62.5	Muslims	0.57	0.05	0.48	0.67
				28.13	61.5	Russian Orthodox people	0.57	0.08	0.40	0.73
#10 (data used by Simon, Eschert, et al., 2019)	Experimental	Students	Germany	21.10	70.6	*Neues Zentrum* ^a^	0.39	0.11	0.16	0.61
#11 (unpublished data)	Experimental	Students	Germany	27.98	50.0	*Neues Zentrum* ^a^	0.59	0.10	0.39	0.79

*Note.* In addition to the features of the requested and preprocessed data (sub)sets, the table shows the estimates (Est.), the standard errors (*SE*s), and the lower (L95%CI) and upper (U95%CI) bounds of the 95% confidence intervals for the standardized regression coefficients that describe the relations between tolerance (dependent variable) and respect (predictor). LGBTs = lesbian, gay, bisexual, and transgender individuals.

a *Neues Zentrum* (English: New Center) is a fictitious German student group used in the cover story for the experiments. It was stated that the group is close to the *Alternative für Deutschland* (AfD, English: Alternative for Germany), which is an existing right-wing party in Germany. For more details about the student group *Neues Zentrum*, see Simon, Eschert, et al. (2019).

According to the DRM, for tolerance toward a particular group to exist, this group has to be an outgroup that is met with disapproval. Because of this, participants had to be excluded if they were members of that particular group or if they did not disapprove of that group. To this end, for each participant, we checked for whether the rating of his or her approval on a 7-point bipolar scale ranging from −3 to 3 (with 0 as midpoint) was equal to or greater than zero. Note that the approval measure assessed approval of what the outgroup stands for (i.e., the members’ beliefs, preferences, and practices). Thereby, it tapped the psychologically or subjectively essential, defining feature of the outgroup as a collective rather than some stereotypical characteristic. It also did not assess any kind of interpersonal disliking of individual outgroup members.

### Coding

The second and last authors coded each data set along multiple dimensions: membership in a numerical minority or majority, social status, type of group, design, analysis model, respect measure, country of residence, sample, mean disapproval, mean age, percentage of women, and number of participants. Interrater agreement was perfect except for the outgroup’s social status and its type of group, where Rater 2 argued that for the outgroup of atheists (disapproved of by Polish Catholics, for example), it was hard to assign one of the predefined codes. Disagreement was resolved via discussion, which finally led to the assignment of missing values.

#### Membership in a numerical minority or majority

We coded each sample and each outgroup as a numerical minority or a numerical majority.

#### Social status

Each sample and each outgroup was coded as a low or high social status group.

#### Type of group

Moreover, both the samples and the outgroups were coded according to whether they were a political group, a religious group, a life-style group, or an ethnic group.

#### Design

Each study was coded as cross-sectional, longitudinal, or experimental.

#### Analysis model

We coded each analysis model according to whether it included one or more covariates in addition to the measures of respect and tolerance, and thus controlled for third variables.

#### Respect measure

Each measure of respect was coded as a single item, a scale score, or a latent variable. We assigned a missing value when respect was manipulated in an experiment rather than measured and the respect variable was a dummy. Notice, however, that in some experiments, after manipulating respect, it was also measured to check for whether its manipulation had been successful. In this case, we assigned a code to this (additional) respect variable depending on the respect measure that was used.

#### Country of residence

We coded each sample as stemming from Germany, Poland, the United States, or Brazil.

#### Sample

The larger research project included samples of Tea Party supporters; Catholics; Protestants; Muslims; Alevis; lesbian, gay, bisexual, and transgender (LGBT) individuals; or university students, which we coded accordingly.

#### Mean age

We computed the mean age for each sample by aggregating across the participants’ age.

#### Percentage of women

For each sample, we computed the percentage of women in the sample as a proxy for the gender distribution.

#### Number of participants

This was the sample size available for analysis (for an explanation of why, in some samples, we computed more than one mean age, percentage of women, or number of participants depending on the specific outgroup, see the “Analytic Approach” section).

These variables were used to identify possible moderators. Our analytic approach proceeded in multiple steps, each of which will be described in detail in the next section.

### Analytic Approach

The analytic approach we employed consisted of two basic steps. First, for each data set, we performed correlation analyses. The resulting correlation matrices were then used as the input of regression analyses to assess respect–tolerance links with standardized regression coefficients. In the second step, the respect–tolerance links were subjected to a meta-analysis (to assess the overall link) or a meta-regression analysis. In the meta-regression analysis, we used a moderator to explain the differences in the respect–tolerance link across data sets.

#### Step 1: Assessing respect–tolerance links

Many data sets contained additional, possibly confounding third variables, including the strength of participants’ approval of the outgroup as well as the sociological variables—age, gender, education, and income. Along with the respect and tolerance variables, these third variables were subjected to correlation analyses, which were computed in R (R Development Core Team, 2016). In these analyses, we computed bivariate correlations between the variables, which built the basis for further analyses. Among these, the correlations with respect differed in the extent to which the measurement error in the respect variable was accounted for. To fully account for this error, we adopted a latent-variable approach and used the R package lavaan (Rosseel, 2012) to model respect by means of an unconstrained measurement model with freely estimated loadings and error variances. Alternatives to the use of a latent-variable approach that are often less reliable are the scale score and the single item, which we also used. Note that the question of whether the choice of, for example, a latent variable instead of a scale score would affect the respect–tolerance link was addressed by performing an analysis of moderating effects in Step 2 of our analytic approach (see more below).

To deal with missing values in the variables, we applied full information maximum likelihood (FIML), which allowed us to use all the information available in the data. This approach handles missing values in variables on the basis of the assumption that the missing values depend on other variables in the data set (missing-at-random assumption; Rubin, 1987). Note also that FIML is recommended by many scholars (e.g., Allison, 2003) and is often applied in psychological research.

Because the correlation analyses resulted in more than one correlation matrix per data set, we adopted a shifting-unit-of-analysis approach (Cooper, 1998). In the first step of this approach, we aggregated correlation matrices that came from the same sample and did not differ with respect to the moderator of interest (see Lipsey & Wilson, 2001). To this end, we used the weighted average (weighted by the number of participants; Hunter & Schmidt, 2004). To illustrate, suppose a data set in which multiple correlation analyses that differed with respect to the outgroup were conducted. To prepare the input for the estimation of the DRM, we aggregated the correlation matrices across these different outgroups. However, when our aim was to investigate whether the outgroup would act as a moderator in the DRM model, we did not aggregate these correlation matrices but subjected them to further analyses separately. This approach minimizes the dependence that is due to the fact that multiple correlation matrices stem from the same sample while preserving all the information relevant for conducting the meta-regression analyses (see O’Mara et al., 2006).

After we aggregated the correlation matrices, we estimated the DRM for each aggregated correlation matrix by fitting multiple regression models with the OpenMx software (Boker et al., 2017). In the basic cross-sectional model, tolerance was regressed only on respect:

(1) $${\mathrm{TOL}}_{i}=\beta_{1}{\mathrm{RESP}}_{i}+r_{i},$$

where TOL_*i*_ is the *i*th person’s tolerance toward an outgroup, RESP_*i*_ is the person’s respect for this group, and *r_i_* are residuals. β_1_ describes the relation between respect and tolerance, and was thus the parameter that was of primary interest in this model. In the longitudinal case, tolerance at time point *t* was predicted by respect at an earlier time point *t* – 1, while controlling for tolerance at that earlier time point (lagged criterion). The model reads:

(2) $${\mathrm{TOL}}_{t,i}=\beta_{1}{\mathrm{RESP}}_{t-1,i}+\beta_{2}{\mathrm{TOL}}_{t-1,i}+r_{i}.$$

In accordance with the procedure used in the published studies, the models could include one or more additional covariates; for example, the participants’ age, gender, education, income, or even approval of the outgroup, which allowed us to preclude a potential confounding by these variables. The question of whether β_1_ would differ as a function of covariates was addressed in Step 2.

Where the model was fit to an aggregated correlation matrix, we used the average sample size $\bar{N}$ (aggregated over different numbers of participants, which could vary from outgroup to outgroup due to the DRM’s requirement that participants must not be members of the group that is being met with disapproval themselves). However, fitting the model to an aggregated correlation matrix was not problem-free. Therefore, before we estimated the model, we first checked whether the correlation matrix was non-positive definite. When this was the case, we applied Yuan and Chan’s (2008) ridge technique to the matrix and passed the “cured” instead of the degenerate matrix to OpenMx. The ridge technique adds a small value *a* to the main diagonal of the singular matrix **R**: *R*^1^ = **R** + *a* **I**, where **I** is the identity matrix (for another application of this technique in a meta-analysis, see Möller et al., 2020). From each fitted DRM, we extracted the standardized regression coefficient (i.e., the relation between respect and tolerance) and its standard error.

#### Step 2: Explaining differences in the respect–tolerance link

To pool the respect–tolerance links, we fit a random-effects model using metaSEM (Cheung, 2015b). Applying the common notation for hierarchical models (e.g., Raudenbush & Bryk, 2002), the random-effects model reads at the first level:

(3) $$Level\;1:\;\;t_{i}=\;\theta_{i}+e_{i},$$

where *t_i_* is the point estimate as derived from the *i*th correlation matrix, θ_*i*_ is the respective true parameter, and *e_i_* is the deviation of the point estimate from the true parameter. At the second level, the following relation holds:

(4) $$Level\;2:\;\;\theta_{i}=\;\theta +u_{i},$$

where θ is the average population parameter, and *u_i_* is the deviation of θ_*i*_ from θ. Unlike the fixed-effects model, this model takes into account the variability across data sets (e.g., Overton, 1998). Notice that the meta-analytic approach of pooling regression coefficients (sometimes also referred to as the parameter-based approach because one or more model parameters are meta-analyzed; see Becker & Wu, 2007; Gasparrini et al., 2012; see also Cheung, 2015a) was chosen here instead of pooling correlations so that comparisons could be made with past tolerance studies, which used the standardized regression coefficient to describe the respect–tolerance link. Moreover, it allowed us to investigate the influence of categorical and, most importantly, continuous moderators (e.g., number of participants) on this link using meta-regression analysis.

To this end, we estimated mixed-effects models for each of the moderators. When a discrete moderator with *m* categories was investigated, we first created *m* – 1 dummy variables, $C_{1},C_{2},\ldots ,C_{m-1}$ , which were then added as predictors to the second level of the model:

(5) $$Level\;2:\;\;\theta_{i}=\theta +\gamma_{1}C_{1,i}+\;\gamma_{2}C_{2,i}+\cdots +\gamma_{m-1}C_{m-1,i}+u_{i},$$

where $\gamma_{k}(k=1,2,\ldots ,m-1)$ is the difference between the category that is coded by *C_k_* and the reference category. For continuous moderators, we first standardized the moderator and then added it as a predictor:

(6) $$Level\;2:\;\;\theta_{i}=\theta +\gamma M_{i}+u_{i},$$

where γ is the slope of the moderator *M_i_*. In addition, meta-regression analysis also allowed us to add additional variables as covariates to the model to control for unwanted confounding by these variables. Recall that some of the moderators and covariates had missing values (see the “Coding” section for an explanation for why the outgroup’s social status had missing values, for example). Unfortunately, estimating the mixed-effects models allowed us to use only complete cases in the explanatory variables, which is why we used listwise deletion in this case.

## Results

To evaluate our main research question, which asked whether respect would predict tolerance on average, we applied the random-effects model as described above. The pooled standardized regression coefficient was 0.25 (*p* < .001), and its 95% confidence interval ranged from 0.16 to 0.34, thereby clearly supporting the outgroup respect–tolerance hypothesis that people’s respect influences their tolerance (see Table 3 for further details about the averaged results).

**Table 3.** Average Results for the Relation Between Respect and Tolerance.

Moderator	Categories	Overall No. of observations used	No. of data sets used	Average No. of observations used	Standardized regression coefficient			
					Est.	*SE*s	L95%CI	U95%CI
No moderator		3,193	11	290	0.25***	0.05	0.16	0.34
Substantive moderators								
Numerical minority or majority (sample)	Minority	2,455	7	351	0.18***	0.04	0.10	0.26
	Majority	738	4	184	0.40***	0.06	0.28	0.52
Numerical minority or majority (outgroup)	Minority	3,366	11	306	0.25***	0.05	0.14	0.36
	Majority	1,735	6	289	0.26***	0.07	0.12	0.41
Social status (sample)	Low	1,151	4	288	0.20**	0.07	0.06	0.34
	High	2,042	7	292	0.29***	0.06	0.18	0.39
Social status (outgroup)	Low	3,050	11	277	0.21**	0.07	0.08	0.34
	High	2,133	7	305	0.22**	0.08	0.06	0.38
Type of group (sample)	Political	562	1	562	0.25^†^	0.14	−0.02	0.53
	Religious	1,921	6	320	0.23***	0.06	0.11	0.34
	Life-style	710	4	178	0.30***	0.08	0.15	0.46
Type of group (outgroup)	Political	3,098	10	310	0.36***	0.05	0.26	0.46
	Religious	2,714	9	302	0.19***	0.05	0.08	0.29
	Life-style	2,708	7	387	0.26***	0.06	0.15	0.38
	Ethnic	185	2	93	−0.34**	0.13	−0.58	−0.09
Methodological moderators								
Design	Cross-sectional	3,119	9	347	0.44***	0.03	0.38	0.50
	Longitudinal	2,769	8	346	0.18***	0.03	0.12	0.24
	Experimental	111	2	56	0.50***	0.09	0.31	0.68
Analysis model	No covariates	4,113	11	374	0.27***	0.05	0.18	0.37
	Covariates included	3,141	11	286	0.25***	0.05	0.16	0.35
Respect measure	Single item	3,203	11	291	0.23***	0.06	0.12	0.34
	Scale score	3,170	11	288	0.28***	0.06	0.17	0.39
	Latent variable	3,206	11	291	0.37***	0.06	0.26	0.48
Additional moderators								
Country of residence	Germany	1,506	6	251	0.25***	0.06	0.14	0.36
	Poland	627	2	313	0.34***	0.09	0.17	0.52
	The United States	827	2	413	0.25**	0.09	0.07	0.42
	Brazil	233	1	233	0.09	0.13	−0.16	0.33
Sample	Tea Party supporters	562	1	562	0.25**	0.08	0.10	0.41
	Catholics	627	2	313	0.35***	0.06	0.23	0.47
	Protestants	743	2	371	0.11^†^	0.06	0.00	0.22
	Muslims	410	1	410	0.28**	0.09	0.11	0.45
	Alevis	141	1	141	0.18^†^	0.11	−0.03	0.39
	LGBTs	599	2	300	0.16**	0.06	0.04	0.29
	University students	111	2	56	0.50***	0.09	0.32	0.67

*Note.* In addition to the estimates (Est.), the table shows the standard errors (*SE*s) and the lower (L95%CI) and upper (U95%CI) bounds of the 95% confidence intervals for the average standardized regression coefficients (aggregated across data sets) of the moderator variables. LGBTs = lesbian, gay, bisexual, and transgender individuals.

† *p* < 1. **p < .01. ***p < .001 (all *p*s two-sided).

We tested the effects of the moderators by computing meta-regression analyses. We proceeded by first analyzing the moderator of interest separately without any further moderators. However, for some moderators, because this analysis was expected to be confounded by other variables, we performed another analysis in which we controlled for these potential confounding variables (e.g., design). The resulting adjusted effects of the moderators were free from any influence from the confounders. Notice also that although we included only the variables that were considered most critical, one might argue that other variables could also act as confounders (e.g., number of participants). However, we did not control for them for three reasons. First and foremost, whereas it is easy to justify why we controlled for design in almost every analysis, it is less obvious to explain how other variables could confound the analyses. For example, it would be unlikely for the number of participants to affect the effects of the moderators. Moreover, the variables analysis model and respect measure could not affect the effect sizes of the moderators because they were virtually uncorrelated (i.e., orthogonal) with all other variables. Third, there were missing values in the outgroup’s social status and type of group variables. Because we used only complete cases in the analyses, including these variables would have led to the (listwise) deletion of cases, and we wanted to avoid doing this so that we could preserve as much of the information as possible (see Zhou et al., 2019 for a similar argument for why variables with missing values were not entered as covariates in their analyses).

To test the effect that each moderator of interest had, we compared two nested models (one unconstrained model with the assumed effect for the moderator of interest vs. one constrained model without it) using the likelihood ratio test (LRT), which is also known as the chi-square difference test. To specify the constrained model, we fixed the moderator’s slopes ( $\gamma_{1},\gamma_{2},\ldots ,\gamma_{m-1}$ for categorical moderators, γ for continuous moderators; see “Analytic Approach” section) to zero. For categorical moderators with more than two categories, we conducted additional pairwise comparisons in addition to this omnibus test of moderating effects to identify significant differences between the categories (see Tables 4 and 5). For all statistical tests, α was set to the nominal 5% level.

**Table 4.** Pairwise Comparisons (Uncontrolled).

Moderator	Comparisons		Difference			
			Est.	*SE*s	L95%CI	U95%CI
Substantive moderators						
Numerical minority or majority (sample)	Minority	– Majority	−0.22**	0.07	−0.08	−0.36
Numerical minority or majority (outgroup)	Minority	– Majority	−0.01	0.09	0.16	−0.19
Social status (sample)	Low	− High	−0.09	0.09	0.09	−0.27
Social status (outgroup)	Low	− High	−0.01	0.11	0.20	−0.22
Type of group (sample)	Political	– Religious	0.03	0.15	0.33	−0.27
		– Life-style	−0.05	0.16	0.27	−0.36
	Religious	– Life-style	−0.08	0.10	0.12	−0.27
Type of group (outgroup)	Political	– Religious	0.17*	0.07	0.32	0.03
		– Life-style	0.10	0.08	0.25	−0.05
		– Ethnic	0.70***	0.14	0.96	0.43
	Religious	– Life-style	−0.07	0.08	0.08	−0.23
		– Ethnic	0.52***	0.14	0.79	0.26
	Life-style	– Ethnic	0.60***	0.14	0.87	0.33
Methodological moderators						
Design	Cross-sectional	– Longitudinal	0.26***	0.04	0.35	0.18
		– Experimental	−0.05	0.10	0.14	−0.24
	Longitudinal	– Experimental	−0.32**	0.10	−0.13	−0.51
Analysis model	No covariates	– Covariates included	0.02	0.07	0.15	−0.11
Respect measure	Single item	– Scale score	−0.05	0.08	0.11	−0.21
		– Latent variable	−0.13^†^	0.08	0.02	−0.29
	Scale score	– Latent variable	−0.08	0.08	0.07	−0.24
Additional moderators						
Country of residence	Germany	− Poland	−0.10	0.11	0.11	−0.30
		– The United States	0.00	0.11	0.21	−0.21
		– Brazil	0.16	0.14	0.43	−0.11
	Poland	– The United States	0.10	0.13	0.34	−0.15
		– Brazil	0.26^†^	0.16	0.56	−0.05
	The United States	– Brazil	0.16	0.15	0.46	−0.14
Sample	Tea Party supporters	– Catholics	−0.10	0.10	0.10	−0.29
		– Protestants	0.15	0.10	0.33	−0.04
		– Muslims	−0.02	0.12	0.20	−0.25
		– Alevis	0.08	0.13	0.33	−0.18
		– LGBTs	0.09	0.10	0.28	−0.10
		–University students	−0.24*	0.12	−0.01	−0.47
	Catholics	– Protestants	0.24**	0.08	0.40	0.08
		–Muslims	0.07	0.11	0.28	−0.14
		– Alevis	0.17	0.12	0.41	−0.07
		− LGBTs	0.18*	0.09	0.35	0.01
		– University students	−0.15	0.11	0.07	−0.36
	Protestants	– Muslims	−0.17	0.10	0.03	−0.37
		– Alevis	−0.07	0.12	0.17	−0.31
		− LGBTs	−0.06	0.08	0.11	−0.22
		– University students	−0.39***	0.11	−0.18	−0.60
	Muslims	– Alevis	0.10	0.14	0.37	−0.17
		– LGBTs	0.11	0.11	0.32	−0.10
		– University students	−0.22^†^	0.12	0.03	−0.46
	Alevis	– LGBTs	0.01	0.12	0.25	−0.23
		– University students	−0.32*	0.14	−0.05	−0.59
	LGBTs	– University students	−0.33**	0.11	−0.12	−0.55

*Note.* In addition to the estimates (Est.), the table shows the standard errors (*SE*s) and the lower (L95%CI) and upper (U95%CI) bounds of the 95% confidence intervals for the mean differences (aggregated across data sets) between the standardized regression coefficients of the moderators’ categories. LGBTs = lesbian, gay, bisexual, and transgender individuals.

† *p* < 1. *p < .05. **p < .01. ***p < .001 (all *p*s two-sided).

**Table 5.** Pairwise Comparisons Controlled for Possibly Confounding Variables.

Moderator	Comparisons		Difference				Confounders
			Est.	*SE*s	L95%CI	U95%CI	
Substantive moderators							
Numerical minority or majority (sample)	Minority	– Majority	−0.18***	−0.04	−0.11	−0.26	Design, social status (sample), type of group (sample)
Numerical minority or majority (outgroup)	Minority	– Majority	−0.08	−0.12	0.15	−0.31	Design, social status (outgroup), cultural background (outgroup)
Social status (sample)	Low	– High	0.19***	−0.04	0.28	0.11	Design, numerical minority or majority (sample), type of group (sample)
Social status (outgroup)	Low	– High	0.07	−0.11	0.28	−0.14	Design, numerical minority or majority (outgroup), type of group (outgroup)
Type of group (sample)	Political	– Religious	0.16***	−0.04	0.24	0.09	Design, numerical minority or majority (sample), social status (sample)
		– Life-style	0.28***	−0.06	0.40	0.16	—”—
	Religious	– Life-style	0.12*	−0.05	0.21	0.02	—”—
Type of group (outgroup)	Political	– Religious	0.08	−0.07	0.22	−0.05	Design, numerical minority or majority (outgroup), social status (outgroup)
		– Life-style	0.06	−0.08	0.22	−0.11	—”—
		– Ethnic	0.67***	−0.13	0.93	0.42	—”—
	Religious	– Life-style	−0.03	−0.07	0.12	−0.17	—”—
		– Ethnic	0.59***	−0.13	0.84	0.34	—”—
	Life-style	– Ethnic	0.62***	−0.13	0.87	0.36	—”—
Methodological moderators							
Design	Cross-sectional	– Longitudinal	0.25***	−0.03	0.30	0.20	Numerical minority or majority (sample), social status (sample), type of group (sample)
		– Experimental	−0.10	–0.09	0.08	−0.29	—”—
	Longitudinal	– Experimental	−0.35***	–0.10	−0.16	−0.54	—”—
Additional moderators							
Country of residence	Germany	− Poland	−0.09^†^	−0.05	0.01	−0.18	Design
		– The United States	−0.06	−0.04	0.03	−0.15	“
		– Brazil	0.12^†^	−0.06	0.23	0.00	“
	Poland	– The United States	0.03	−0.06	0.13	−0.08	“
		– Brazil	0.20**	−0.07	0.34	0.07	“
	The United States	– Brazil	0.18**	−0.06	0.30	0.05	“

*Note.* In addition to the estimates (Est.), the table shows the standard errors (*SE*s) and the lower (L95%CI) and upper (U95%CI) bounds of the 95% confidence intervals for the unconfounded mean differences (adjusted by confounders) between the standardized regression coefficients of the moderators’ categories.

† *p* < 1. *p < .05. **p < .01. ***p < .001 (all *p*s two-sided). —”— and “ indicate that the same confounders were controlled for as in the previous comparison.

In reporting the results, we begin with the results for the research questions. In the next step, we present the results of the exploratory analyses.

### Membership in a Numerical Minority or Majority

When the two models (unconstrained vs. constrained) were compared in the first meta-regression analysis (without any additional moderators), the LRT with one degree of freedom yielded a value of 7.01, which was significant (*p* < .01) and thus clearly supported the speculation that the sample’s membership in a numerical minority or majority would affect the respect–tolerance link. This effect was considered large because its size of *f* ^2^ = 1.20 exceeded the critical value of 0.35 for large effects. We used Cohen’s *f* ^2^ here because Selya et al. (2012; see also Lorah, 2018) suggested that it would quantify the effects in hierarchical models, and common guidelines for its interpretation exist (see Cohen, 1988). Moreover, as can be seen in Table 4, the difference in the respect–tolerance link between the two categories “numerical minority” and “numerical majority” was −0.22 and significant (*p* < .01), suggesting that participants belonging to a numerical majority exhibited significantly stronger relations between respect and tolerance than those belonging to a numerical minority did. However, it was reasonable to assume that this analysis was to some extent confounded by other moderators, most likely the research design.

Moreover, we assumed that the sample’s social status and its type could also act as confounders. Therefore, in the second analysis, we controlled for these variables to check for whether a moderating effect of the sample’s membership in a numerical minority or majority would still occur when confounding by these variables was precluded. However, the finding proved to be robust as indicated by a value of 15.46 of the LRT statistic with one degree of freedom, which was significant (*p* < .001). Also, the comparison confirmed that numerical majorities showed a significantly stronger link than numerical minorities did (*p* < .001; see Table 5).

However, we did not find any significant effect of membership in a numerical minority or majority for the outgroup. This finding held in the analysis that did not include the confounding variables, design, social status, or type of outgroup, Δχ^2^(1) = 0.02, and when these confounders were included as covariates, Δχ^2^(1) = 0.46.

### Social Status

To test for a moderating effect of the sample’s social status in the first analysis (without any other moderators), we used an LRT with one degree of freedom. The LRT statistic was 0.88 and failed to reach significance. In the second analysis, we added three moderators as covariates to control for unwanted confounding: design, the sample’s membership in a numerical minority or majority, and its type. The LRT with one degree of freedom yielded a much larger value of 16.42, which was significant (*p* < .001). Contrary to our speculation, the difference in the respect–tolerance link between the two categories “low” and “high” social status was significant and positive (0.19, *p* < .001), which indicated that participants belonging to a low-status group showed a significantly stronger link than those belonging to a high-status group.

We did not find any significant effect of the outgroup’s social status in the first analysis, Δχ^2^(1) = 0.01, nor did we find one in the second analysis, Δχ^2^(1) = 0.38, when we included design, membership in a numerical minority or majority, and type of outgroup as covariates.

### Type of Group

Model comparison with two degrees of freedom yielded a value of 0.61 in the first analysis (without any other moderators), which was not significant. However, when possible confounders (design, membership in a numerical minority or majority, and the social status of the sample) were included in the second analysis, the LRT statistic with two degrees of freedom was 16.35 and thus supported the significant impact of the type of sample on the respect–tolerance link (*p* < .001). This effect was large in terms of the suggested cutoffs (i.e., *f* ^2^ > 0.35). We found significant differences between the category “political” group and the other two types of groups. The political samples showed stronger relations between respect and tolerance than the religious and the life-style samples (*p*s < .001), and the religious samples showed stronger relations than the life-style samples (*p* < .05) (see Table 5).

A significant and large effect of the type of outgroup was observed in the first analysis, Δχ^2^(2) = 3.09, *p* < .001, *f* ^2^ = 1.16, with the political outgroups exhibiting a stronger respect–tolerance link than the religious (*p*s < .05) or ethnic (*p* < .001) outgroups (see Table 4); the religious outgroups exhibiting a stronger link than the ethnic outgroups (*p* < .001); and the life-style outgroups exhibiting a stronger link than the ethnic outgroups (*p* < .001). However, although the moderating effect remained significant and large in the second analysis in which we controlled for design, the outgroup’s membership in a numerical minority or majority, and the outgroup’s social status, only the comparisons with the ethnic outgroups were significant (all *p*s < .001).

### Design

In the first analysis (without any other moderators), the result of the LRT with two degrees of freedom for the moderating effect of design was 21.42, which indicated a significant effect (*p* < .001), thus supporting the expectation that the design would affect the respect–tolerance link. With a size of *f* ^2^ = 3.24, this effect was considered large, and pairwise comparisons indicated that the respect–tolerance link was significantly stronger for cross-sectional than for longitudinal designs (*p* < .001) and significantly stronger for experimental than for longitudinal designs (*p* < .01).

To test the robustness of these results, we controlled for membership in a numerical minority or majority, social status, and the type of sample in the second analysis. The LRT with two degrees of freedom yielded a value of 35.81, indicating significance (*p* < .001). Moreover, of the pairwise comparisons, the difference between the cross-sectional and longitudinal designs (Δ = 0.25) and the difference between the experimental and longitudinal designs (Δ = 0.35) were both significant (both *p*s < .001), with the cross-sectional designs showing stronger relations between respect and tolerance than the longitudinal designs, and the experimental designs showing stronger relations than the longitudinal designs.

### Analysis Model

To test for whether the analysis model affected the respect–tolerance link, we conducted an LRT with one degree of freedom. Recall that we distinguished between models with one or more covariates (e.g., where approval of the outgroup was controlled for) and models without covariates in which tolerance was predicted only by respect. The test yielded a value of 0.09, which was not significant. Notice also that the analysis model was among the variables that could not be confounders for statistical reasons (because they were virtually orthogonal to all other variables). This also means that if the analysis model served as the moderator in the moderator analysis, adding other variables as covariates would not alter the size of the moderating effect. Therefore, we did not conduct another analysis in which we controlled for these variables.

### Respect Measure

We conducted another analysis in which we tested the role of the respect measure as a possible moderator but did not find any significant effect, Δχ^2^(2) = 2.71. However, because we had specific directed hypotheses on the differences between the measures with regard to the size of the respect–tolerance link, we considered the pairwise comparisons to be the critical tests. Table 4 shows that despite the absence of evidence of an overall effect of the respect measure, we found support for our hypothesis that the use of the latent variable would yield larger sizes of this link than the use of a single item would. Note that because the hypothesis was directed, we considered marginal significance (i.e., *p* < .1, two-sided) to be indicative of a reliable difference in this case. However, although the latent variables tended to yield a stronger link than the scale scores, this difference was not reliable as was the difference between the scale scores and the single items (*p*s > .1, two-sided). Note also that for the same reason as for when the analysis model was the moderator, we did not conduct a second analysis to control for possible confounders.

In an additional analysis, we investigated whether the five respect items in the single-item category were differentially related to tolerance and which of the five respect items in the single-item category explained the most variance. The LRT statistic with four degrees of freedom was 16.11 and significant (*p* < .01). The size was *f* ^2^ = 1.10 and was thus rather large. However, the pairwise comparisons suggested that significant differences (all *p*s < .05) only existed between items that were used in the correlational studies and items that were exclusively used in the experiments (Studies 10 and 11), making it difficult to attribute these differences solely to the items.

Next, we report the results for the additional moderators, for which we did not have any hypotheses and which were thus purely exploratory in nature.

### Country of Residence

The LRT for the moderating influence of country of residence had three degrees of freedom and yielded a value of 2.35 in the first analysis (without any other moderators), which was not significant. When we controlled for design in the second analysis, the LRT statistic with three degrees of freedom was 8.54, which indicated significance (*p* < .05). Applying the common cutoffs, the effect could be considered large (*f* ^2^ = 1.15).

As revealed by pairwise comparisons, participants from Poland and the United States provided stronger respect–tolerance links than those from Brazil (*p*s < .01). Moreover, we found a marginally significant difference between Germany and Poland (*p* < .1), where the link was stronger in Poland than in Germany. We also found a difference between Germany and Brazil, where the link was stronger in Germany than in Brazil.

### Sample

We conducted a model comparison with six degrees of freedom. The associated LRT statistic was 11.63, which failed to reach significance, indicating that at most, there was a tendency for the samples to vary with regard to the respect–tolerance link. Additional pairwise comparisons showed that university students provided significantly stronger respect–tolerance relations than Tea Party supporters (*p* < .05), Protestants (*p* < .001), Muslims (*p* < .1), Alevis (*p* < .05), and LGBTs (*p* < .01). Moreover, we found that the link was significantly stronger for Catholics than for Protestants (*p*s < .01) and LGBTs (*p* < .05). However, it should be noted that because the moderating effect of the sample was mainly driven by the differences between the students and all other samples and this group participated exclusively in the experiments, the result should be interpreted with caution.

### Mean Age

A model comparison with one degree of freedom indicated no significant effect of the mean age of the sample on the respect–tolerance link. This finding held in the first analysis without any other moderators, Δχ^2^(1) = 1.46, and the second analysis with design as the covariate, Δχ^2^(1) = 0.26.

### Percentage of Women

The LRT statistic of the test of the moderating influence of the gender distribution had one degree of freedom. The value was 5.23 and significant in the first analysis (without any other moderators; *p* < .05). The size of the effect was *f* ^2^ = 0.05 and thus above the cutoff of 0.02 for small effects. However, when we controlled for design in the second analysis, a significant effect was no longer observed.

### Number of Participants

We found a significant moderating influence of the number of participants in the first analysis, without any other moderators, Δχ^2^(1) = 5.06, *p* < .05. However, because of its size of *f* ^2^ = 0.05, this effect was small in terms of the suggested cutoffs. When we controlled for design in the second analysis, a marginally significant effect was still observed, with the larger samples showing a stronger respect–tolerance link.

### Summary

The most important findings can be summarized as follows. We found clear support for our main hypothesis that the relation between respect and tolerance would exist on average and be substantial. Moreover, we also found evidence for some moderating influences. First and foremost, the sample’s characteristics played a moderating role in the relation between respect and tolerance. More specifically, respect was more predictive of tolerance among members of numerical majorities than minorities, and—unexpectedly—it was more predictive among members of low-status groups than high-status groups. Moreover, we found that members of religious groups exhibited a stronger respect–tolerance link than members of life-style groups did and that members of political groups exhibited an even stronger link than members of religious or life-style groups did.

Second, we found some evidence that the methodology played a moderating role. Cross-sectional studies and experiments showed stronger relations between respect and tolerance than longitudinal studies, which was not very surprising. For example, in experimental designs, respect is actively manipulated and is thus under the control of the experimenter as is the extent to which confounding variables are allowed to interfere. But in longitudinal designs, the effect depends on naturally occurring processes (i.e., processes that prompt changes in the level of respect). Moreover, we found that the respect–tolerance link was rather robust against variations in the model employed for data analysis and in the type of measure used to assess respect. We did not find evidence that adding one or more covariates to the DRM affected the respect–tolerance link, suggesting that alternative explanations in terms of these variables did not apply and that the DRM in its simplest form (i.e., including only respect and tolerance variables) need not be refined substantially. Also, the respect–tolerance link was stronger for the latent variable than for the single item, thereby pointing to the usefulness of the latent-variable approach when respect is assessed with multiple items. However, it should be noted that the results did not suggest that the latent-variable approach was superior to the scale score, which encourages the view that the scale score could be an attractive alternative to the latent-variable approach in social-psychological research, particularly in situations in which the adoption of a latent-variable approach is troublesome (e.g., in small sample contexts in which convergence issues are likely to occur).

## Discussion

Motivated by the topic’s high relevance to society, we evaluated whether respect for others as equals fosters tolerance toward others who are met with disapproval. To this end, we determined the pooled strength of this relation across a comprehensive set of empirical studies. Overall, respect was a significant, substantial predictor of tolerance. Moreover, the relation was influenced by a number of moderators in ways that can be informative for future research. We discuss this further below, where we also discuss the specific meaning of the observed moderations as well as their relevance to the model and the ways in which they may contribute to the model’s further development.

Methodologically, we demonstrated the use of meta-analytic techniques, which we employed to synthesize a comprehensive collection of existing data sets. We did this in a manner similar to systematic literature reviews. However, our work differed from most of these reviews. Systematic reviews often involve a large number of different conceptualizations of the constructs, which can blur the relations between these and other constructs. By using the data from a large multistudy research project, we ensured that the conceptualizations (e.g., where respect was defined as viewing others as equals) were consistent across the different studies that were included in our analysis. This provided us with a more robust test of the outgroup respect–tolerance hypothesis. We found that the relation between respect and tolerance was, on average, positive and substantial, thereby supporting our hypothesis. Thus, we view the use of data from a larger project as an advantage of the present work because interpretational ambiguities due to different conceptualizations of the constructs did not emerge.

Finally, to guarantee transparency, we attempted to be as precise as possible with respect to the exact procedures we employed, and we were clear about which of the analyses were conducted to test hypotheses and which of them were exploratory. Besides these strengths of the present work, the following limitations should also be mentioned.

### Limitations

Most of the data used in the present study were borrowed from correlational studies, which were still vulnerable to unwanted confounding by variables that were not measured in the respective studies. Experiments, which are designed to control for third variables by means of randomization, have to date been rare so far in research on the respect–tolerance link. Therefore, some caution is in order when interpreting our main finding because, strictly speaking, it does not present conclusive evidence of the notion that respect is not just a correlate of tolerance but is rather a cause.

Although our analytic strategy met the requirements of best practice, some of the methodological decisions may be controversial and should be discussed further. First, the parameter-based approach used in the present work has been criticized in the past (e.g., Cheung, 2015a). However, the main criticism of it does not apply here because we did not face the problem of incomplete information when fitting the analysis models. Each data set included one measure of respect in addition to tolerance, which allowed us to estimate the correlation between these variables and thus also the DRM in its simplest form (i.e., the model with tolerance as the dependent variable and respect as the predictor).

Second, to address the dependencies across the multiple effect sizes, we applied the shifting-units-of-analysis approach (Cooper, 1998), which is certainly not without weaknesses (e.g., several steps need to be performed in this analysis instead of just one step). On the contrary, it can be very useful in practice (see Lipsey & Wilson, 2001; see also O’Mara et al., 2006, for an example application of this approach). It should also be noted that there are alternative approaches such as three-level meta-analyses (Cheung, 2014). Although each approach accounts for the dependencies across the effect sizes, they differ with regard to their assumptions (see Cheung, 2019, for a discussion of these approaches). Future research could apply both approaches (or a combination thereof) to check for whether the findings are robust (i.e., they do not change) against the use of different approaches.

Third, missing values were almost completely absent from the moderation analyses except for some moderators, where the ratio of missing values was still extremely rare. In the meta-regression analyses involving these variables, we used only complete cases (i.e., listwise deletion). Alternatively, we could have applied FIML. Not only does FIML handle missing values in dependent variables (e.g., missing effect sizes), but it also allows for the handling of missing values in moderators. However, to cope with missing values in moderators, additional assumptions must be made. For example, missing values must be assumed to be missing at random and—to make this assumption plausible—additional variables on which the missing values depend must be included (e.g., further study features). However, recall that the missing values were partly due to the raters’ decision not to assign any predefined code (see the “Coding” section). Thus, it is questionable whether the correct use of FIML would have been feasible in the present study because it was hard to see which variables the missing values depended on.

Moreover, in our case, in which the effect sizes were fully observed and the missing values in the moderators were extremely rare, we did not consider FIML to be noticeably superior to listwise deletion. Note, however, that we employed FIML successfully in the preprocessing of the data, where the missing values in the variables were more of an issue.

Fourth, we focused on the main effects of the moderators. However, it would be interesting to also investigate interactions between them. For example, future research could study the interplay between the sample’s and the outgroup’s characteristics. We believe that this interplay can be best studied with the help of experiments, which allow researchers to vary these characteristics in a systematic way.

### Directions for Theory Development and Future Empirical Research

The mechanism that the DRM is based on is straightforward. The assumption is that outgroups can be recognized and respected as equals, which then facilitates tolerance toward them, even from others who disapprove of their ways of life. The distinct contribution of the present work is the comprehensive test of the robustness of the respect–tolerance link and our endeavor to uncover possible moderators that can inform and guide the further development of the DRM but also future research on tolerance more generally. Of the many novel empirical findings we reported, we wish to highlight the ones that appear particularly promising to us with regard to their potential to contribute to theory development and corresponding empirical research.

First, we obtained empirical support for our speculation that members of numerical majority groups show stronger respect–tolerance links than members of numerical minority groups do. We had intuited that members of majority groups would see themselves as relatively more prototypical representatives of society with a greater need of a good reason to tolerate the outgroup. Respect for outgroup members should be such a reason. Hence, the more respect majorities or high-status groups actually grant outgroup members, the stronger their reason for tolerance. This account may also hold for our observation that members of religious groups evinced a stronger respect–tolerance link than members of (presumably less obliging) life-style groups.

Another—not necessarily competing but possibly complementary—account comes into view when we turn to the particularly strong link observed for members of political groups compared with both religious and life-style groups. The political sphere—at least in modern democratic societies that emphasize mutual respect for equal fellow citizens—should be a particularly conducive medium for the operation of the respect–tolerance link. From a social-psychological perspective, the cognitive representation of society as the common (higher level) social and political entity comprising members of many different (lower level) groups may well be the decisive factor here. Heterogeneity is a characteristic of modern-day societies (Durkheim, 1997), and the cognitive representation of a society as a heterogeneous superordinate ingroup should imbue the pivotal concept of respect for others as equals with a particular meaning. That is, it should shift its meaning from a narrow understanding of respect for others as “identical” equals as found in homogeneous groups to the wider understanding of respect for others as “different” equals—”different” owing to their membership in different (lower level) groups, “equal” owing to their membership in the same heterogeneous society (Simon, 2020). Whereas the narrow understanding of respect for others as identical equals would be burdened by expectations of conformity, which in turn strain the respect–tolerance link, the wider understanding of respect for others as different equals should, on the contrary, be quite compatible with self–other differences so that respect can easily and strongly be linked up with tolerance. The cognitive-representation account can also contribute to the explanation of the stronger respect–tolerance link observed among majority members that we already discussed above. Relative to minority members, majority members tend to construe their ingroup as a more heterogeneous group (Simon, 1992). If this tendency is combined with the tendency to project the image of one’s ingroup onto society as a whole (Wenzel et al., 2007), the foundation would be laid for a strong respect–tolerance link. Moreover, our observation that members of low-status groups produced a stronger respect–tolerance link than members of high-status groups could be another case in point here. It stands to reason that members of low-status groups are particularly motivated to see society as a heterogeneous entity with heterogeneity signaling openness and encouraging social mobility aspirations. In any case, if confirmed in future research, the cognitive-representation account could turn out to be a particularly powerful explanation because it integrates the specification of a moderator variable (homogeneous vs. heterogeneous cognitive representation of society) with the further specification of the mechanism driving the respect–tolerance link (respect for others as different rather than identical equals).

At this point, we are far from claiming that we are already able to provide a conclusive and integrated theoretical explanation for our numerous empirical observations. Still, these observations challenged us to engage in further theoretical elaboration, and we see additional value in the fact that they pointed us (and hopefully other researchers as well) toward important open questions concerning the role of respect in tolerance. Among these are questions about why ethnic outgroups seem to be particularly hard cases with regard to the emergence of a strong respect–tolerance link and what underlies the differences observed along the national and/or cultural (i.e., country-of-residence) dimensions. Of course, a reliable answer, especially to the latter question, will require future research to employ representative sampling that goes beyond the scope of the research presented in this article.

Finally, despite the complexity of the results and some open questions, there should be no doubt that the present work also has a clear and positive message with important practical implications. People are indeed capable of developing tolerance toward outgroups, without necessarily having to give up their disapproval of these groups. Of course, much depends on whether we are willing to respect each other as equals and therefore depends on the appropriate social and political arrangements that will help people give mutual respect to each other as equals. But once people experience such respect, a positive reciprocity mechanism will likely be set in motion with the potential to help develop tolerant, peaceful, and possibly also cooperative social relationships across group boundaries (see Reininger et al., 2020; Schaefer & Simon, 2019; Simon & Schaefer, 2018).

## Conclusion

To conclude, the outgroup respect–tolerance hypothesis that we scrutinized in the present work was derived from the DRM, which offers one step toward a broader social-psychological theory of intergroup conflict (Simon, 2020). An important challenge for future research is to further develop the model and its connections to other aspects of intergroup relations (e.g., politicization and polarization). We hope that the work presented in this article will serve as a source of inspiration for such research and further theory building as well as an instructive illustration of how (re)analyzing and synthesizing results from multiple data sets coming from a larger research project can contribute to such an important endeavor.

## Supplemental Material

sj-docx-1-psp-10.1177_01461672211024422 – Supplemental material for Does Respect Foster Tolerance? (Re)analyzing and Synthesizing Data From a Large Research Project Using Meta-Analytic Techniques Click here for additional data file. Supplemental material, sj-docx-1-psp-10.1177_01461672211024422 for Does Respect Foster Tolerance? (Re)analyzing and Synthesizing Data From a Large Research Project Using Meta-Analytic Techniques by Steffen Zitzmann, Lukas Loreth, Klaus Michael Reininger and Bernd Simon in Personality and Social Psychology Bulletin

## Notes

1 Multiple tolerance measures were not included in the present study because in all but two of the data sets used here, tolerance was assessed with only a single item.
