# Supplementary material for: Does Respect Foster Tolerance? (Re)analyzing and Synthesizing Data From a Large Research Project Using Meta-Analytic Techniques
Source: Pers Soc Psychol Bull. 2021 Jun 19;48(6):823–43. doi: 10.1177/01461672211024422 (PMC9121536; doi:10.1177/01461672211024422)
Supplement: sj-docx-1-psp-10.1177_01461672211024422 – Supplemental material for Does Respect Foster Tolerance? (Re)analyzing and Synthesizing Data From a Large Research Project Using Meta-Analytic Techniques [file sj-docx-1-psp-10.1177_01461672211024422.docx]

**Muslims in Germany (Study 1)**

The following items were measured at all time points:

**Approval or Disapproval of Christians, Jews, Atheists, Homosexuality and Feminism**

|  | sehr schlecht -3 | ziemlich schlecht -2 | etwas schlecht -1 | neutral 0 | etwas gut +1 | ziemlich gut +2 | sehr gut +3 | kann oder will ich nicht beantworten |
| --- | --- | --- | --- | --- | --- | --- | --- | --- |
| Ist das Christentum etwas Schlechtes oder etwas Gutes? | 🞆 | 🞆 | 🞆 | 🞆 | 🞆 | 🞆 | 🞆 | 🞆 |
| Ist das Judentum etwas Schlechtes oder etwas Gutes? | 🞆 | 🞆 | 🞆 | 🞆 | 🞆 | 🞆 | 🞆 | 🞆 |
| Ist ein Leben ohne Glauben an Gott etwas Schlechtes oder etwas Gutes? | 🞆 | 🞆 | 🞆 | 🞆 | 🞆 | 🞆 | 🞆 | 🞆 |
| Ist Homosexualität etwas Schlechtes oder etwas Gutes? | 🞆 | 🞆 | 🞆 | 🞆 | 🞆 | 🞆 | 🞆 | 🞆 |
| Ist Feminismus etwas Schlechtes oder etwas Gutes? | 🞆 | 🞆 | 🞆 | 🞆 | 🞆 | 🞆 | 🞆 | 🞆 |

**Respect Measurement for Christians, Jews, Atheists, Homosexuality and Feminism**

**Direct respect measurement.**

Welche Behandlung verdienen Ihrer Meinung nach die folgenden Gruppen?

|  | stimme nicht zu 0 | stimme wenig zu 1 | stimme mittelmäßig zu 2 | stimme ziemlich zu 3 | stimme völlig zu 4 | kann oder will ich nicht beantworten |
| --- | --- | --- | --- | --- | --- | --- |
| Christen in Deutschland verdienen Respekt. | 🞆 | 🞆 | 🞆 | 🞆 | 🞆 | 🞆 |
| Juden in Deutschland verdienen Respekt. | 🞆 | 🞆 | 🞆 | 🞆 | 🞆 | 🞆 |
| Menschen ohne Glauben in Deutschland verdienen Respekt. | 🞆 | 🞆 | 🞆 | 🞆 | 🞆 | 🞆 |
| Homosexuelle in Deutschland verdienen Respekt. | 🞆 | 🞆 | 🞆 | 🞆 | 🞆 | 🞆 |
| Feministinnen in Deutschland verdienen Respekt. | 🞆 | 🞆 | 🞆 | 🞆 | 🞆 | 🞆 |

**Equality recognition measurement.**

Welche Behandlung verdienen Ihrer Meinung nach die folgenden Gruppen?

|  | stimme nicht zu 0 | stimme wenig zu 1 | stimme mittelmäßig zu 2 | stimme ziemlich zu 3 | stimme völlig zu 4 | kann oder will ich nicht beantworten |
| --- | --- | --- | --- | --- | --- | --- |
| Christen in Deutschland verdienen Behandlung als gleichwertige Mitbürger. | 🞆 | 🞆 | 🞆 | 🞆 | 🞆 | 🞆 |
| Juden in Deutschland verdienen Behandlung als gleichwertige Mitbürger. | 🞆 | 🞆 | 🞆 | 🞆 | 🞆 | 🞆 |
| Menschen ohne Glauben in Deutschland verdienen Behandlung als gleichwertige Mitbürger. | 🞆 | 🞆 | 🞆 | 🞆 | 🞆 | 🞆 |
| Homosexuelle in Deutschland verdienen Behandlung als gleichwertige Mitbürger. | 🞆 | 🞆 | 🞆 | 🞆 | 🞆 | 🞆 |
| Feministinnen in Deutschland verdienen Behandlung als gleichwertige Mitbürger. | 🞆 | 🞆 | 🞆 | 🞆 | 🞆 | 🞆 |

**Tolerance Measurement**

Sollen die folgenden Gruppen in Deutschland so leben dürfen, so wie die jeweilige Gruppe es möchte?

|  | nein, keinesfalls -2 | -1 | 0 | +1 | ja, unbedingt +2 | kann oder will ich nicht beantworten |
| --- | --- | --- | --- | --- | --- | --- |
| Christen in Deutschland | 🞆 | 🞆 | 🞆 | 🞆 | 🞆 | 🞆 |
| Juden in Deutschland | 🞆 | 🞆 | 🞆 | 🞆 | 🞆 | 🞆 |
| Menschen ohne Glauben in Deutschland | 🞆 | 🞆 | 🞆 | 🞆 | 🞆 | 🞆 |
| Homosexuelle in Deutschland | 🞆 | 🞆 | 🞆 | 🞆 | 🞆 | 🞆 |
| Feministinnen in Deutschland | 🞆 | 🞆 | 🞆 | 🞆 | 🞆 | 🞆 |

**Protestants in Brazil (Study 2)**

The following items were measured in a cross-sectional survey:

**Approval or Disapproval of Catholics, Afro-Brazilian, Atheists, Homosexuality and Feminism**

|  | Muito ruim -3 | Bem ruim -2 | Meio ruim -1 | Neutro 0 | Meio bom +1 | Bem bom +2 | Muito bom +3 | Não sei ou não quero responder |
| --- | --- | --- | --- | --- | --- | --- | --- | --- |
| O catolicismo é algo ruim ou algo bom? | 🞆 | 🞆 | 🞆 | 🞆 | 🞆 | 🞆 | 🞆 | 🞆 |
| As religiões afro-brasileiras são algo ruim ou algo bom? | 🞆 | 🞆 | 🞆 | 🞆 | 🞆 | 🞆 | 🞆 | 🞆 |
| Uma vida sem fé em Deus é algo ruim ou algo bom? | 🞆 | 🞆 | 🞆 | 🞆 | 🞆 | 🞆 | 🞆 | 🞆 |
| A homossexualidade é algo ruim ou algo bom? | 🞆 | 🞆 | 🞆 | 🞆 | 🞆 | 🞆 | 🞆 | 🞆 |
| O feminismo é algo ruim ou algo bom? | 🞆 | 🞆 | 🞆 | 🞆 | 🞆 | 🞆 | 🞆 | 🞆 |

**Respect Measurement for Catholics, Afro-Brazilian, Atheists, Homosexuality and Feminism**

**Direct respect measurement.**

Eu respeito as seguintes pessoas.

|  | De modo nenhum 0 | Pouco 1 | Nem muito, nem pouco 2 | Bastante  3 | Completamente 4 | Não sei ou não quero responder |
| --- | --- | --- | --- | --- | --- | --- |
| Católicos no Brasil | 🞆 | 🞆 | 🞆 | 🞆 | 🞆 | 🞆 |
| Integrantes de religiões afro-brasileiras no Brasil | 🞆 | 🞆 | 🞆 | 🞆 | 🞆 | 🞆 |
| Pessoas sem crença no Brasil | 🞆 | 🞆 | 🞆 | 🞆 | 🞆 | 🞆 |
| Homossexuais no Brasil | 🞆 | 🞆 | 🞆 | 🞆 | 🞆 | 🞆 |
| Feministas no Brasil | 🞆 | 🞆 | 🞆 | 🞆 | 🞆 | 🞆 |

**Equality recognition measurement.**

Eu vejo as seguintes pessoas como cidadãos de mesmo valor que todos os outros.

|  | De modo nenhum 0 | Pouco 1 | Nem muito, nem pouco 2 | Bastante  3 | Completamente 4 | Não sei ou não quero responder |
| --- | --- | --- | --- | --- | --- | --- |
| Católicos no Brasil | 🞆 | 🞆 | 🞆 | 🞆 | 🞆 | 🞆 |
| Integrantes de religiões afro-brasileiras no Brasil | 🞆 | 🞆 | 🞆 | 🞆 | 🞆 | 🞆 |
| Pessoas sem crença no Brasil | 🞆 | 🞆 | 🞆 | 🞆 | 🞆 | 🞆 |
| Homossexuais no Brasil | 🞆 | 🞆 | 🞆 | 🞆 | 🞆 | 🞆 |
| Feministas no Brasil | 🞆 | 🞆 | 🞆 | 🞆 | 🞆 | 🞆 |

**Tolerance Measurement**

No Brasil os seguintes grupos deveriam ter o direito de viver como os seus integrantes gostariam?

|  | Não, de maneira nenhuma -2 | -1 | 0 | +1 | Sim, absolutamente +2 | Não sei ou não quero responder |
| --- | --- | --- | --- | --- | --- | --- |
| Católicos no Brasil | 🞆 | 🞆 | 🞆 | 🞆 | 🞆 | 🞆 |
| Integrantes de religiões afro-brasileiras no Brasil | 🞆 | 🞆 | 🞆 | 🞆 | 🞆 | 🞆 |
| Pessoas sem crença no Brasil | 🞆 | 🞆 | 🞆 | 🞆 | 🞆 | 🞆 |
| Homossexuais no Brasil | 🞆 | 🞆 | 🞆 | 🞆 | 🞆 | 🞆 |
| Feministas no Brasil | 🞆 | 🞆 | 🞆 | 🞆 | 🞆 | 🞆 |

**Protestants in Germany (Study 3)**

The following items were measured at all time points.

**Approval or Disapproval of Catholics, Islam, Atheists and Homosexuality**

|  | sehr schlecht -3 | ziemlich schlecht -2 | etwas schlecht -1 | neutral 0 | etwas gut +1 | ziemlich gut +2 | sehr gut +3 | kann oder will ich nicht beantworten |
| --- | --- | --- | --- | --- | --- | --- | --- | --- |
| Ist der Katholizismus etwas Schlechtes oder etwas Gutes? | 🞆 | 🞆 | 🞆 | 🞆 | 🞆 | 🞆 | 🞆 | 🞆 |
| Ist der Islam etwas Schlechtes oder etwas Gutes? | 🞆 | 🞆 | 🞆 | 🞆 | 🞆 | 🞆 | 🞆 | 🞆 |
| Ist ein Leben ohne Glauben an Gott etwas Schlechtes oder etwas Gutes? | 🞆 | 🞆 | 🞆 | 🞆 | 🞆 | 🞆 | 🞆 | 🞆 |
| Ist Homosexualität etwas Schlechtes oder etwas Gutes? | 🞆 | 🞆 | 🞆 | 🞆 | 🞆 | 🞆 | 🞆 | 🞆 |

**Respect Measurement for Catholics, Islam, Atheists and Homosexuality**

**Direct respect measurement.**

Ich respektiere die folgenden Personen.

|  | gar nicht 0 | wenig 1 | mittelmäßig 2 | ziemlich 3 | völlig  4 | kann oder will ich nicht beantworten |
| --- | --- | --- | --- | --- | --- | --- |
| Katholiken in Deutschland | 🞆 | 🞆 | 🞆 | 🞆 | 🞆 | 🞆 |
| Muslime in Deutschland | 🞆 | 🞆 | 🞆 | 🞆 | 🞆 | 🞆 |
| Menschen ohne Glauben in Deutschland | 🞆 | 🞆 | 🞆 | 🞆 | 🞆 | 🞆 |
| Homosexuelle in Deutschland | 🞆 | 🞆 | 🞆 | 🞆 | 🞆 | 🞆 |

**Equality recognition measurement.**

Ich betrachte die folgenden Personen als gleichwertige Mitbürger*innen.

|  | gar nicht 0 | wenig 1 | mittelmäßig 2 | ziemlich 3 | völlig  4 | kann oder will ich nicht beantworten |
| --- | --- | --- | --- | --- | --- | --- |
| Katholiken in Deutschland | 🞆 | 🞆 | 🞆 | 🞆 | 🞆 | 🞆 |
| Muslime in Deutschland | 🞆 | 🞆 | 🞆 | 🞆 | 🞆 | 🞆 |
| Menschen ohne Glauben in Deutschland | 🞆 | 🞆 | 🞆 | 🞆 | 🞆 | 🞆 |
| Homosexuelle in Deutschland | 🞆 | 🞆 | 🞆 | 🞆 | 🞆 | 🞆 |

**Tolerance Measurement**

Sollen die folgenden Gruppen in Deutschland so leben dürfen, wie deren Mitglieder es möchten?

|  | nein, keinesfalls -2 | -1 | 0 | +1 | ja, unbedingt +2 | kann oder will ich nicht beantworten |
| --- | --- | --- | --- | --- | --- | --- |
| Katholiken in Deutschland | 🞆 | 🞆 | 🞆 | 🞆 | 🞆 | 🞆 |
| Muslime in Deutschland | 🞆 | 🞆 | 🞆 | 🞆 | 🞆 | 🞆 |
| Menschen ohne Glauben in Deutschland | 🞆 | 🞆 | 🞆 | 🞆 | 🞆 | 🞆 |
| Homosexuelle in Deutschland | 🞆 | 🞆 | 🞆 | 🞆 | 🞆 | 🞆 |

**Tea Party Supporters (Study 4)**

The following items were measured at all time points:

**Approval or Disapproval of Islam, Homosexuality, Conservatives and Liberals**

Do you regard the following beliefs and practices as something **good** or something **bad**?

|  | clearly bad -3 | somewhat bad -2 | slightly bad -1 | neutral 0 | slightly good +1 | somewhat good +2 | clearly good +3 | I cannot  or do not  want to  answer |
| --- | --- | --- | --- | --- | --- | --- | --- | --- |
| Islam? | 🞆 | 🞆 | 🞆 | 🞆 | 🞆 | 🞆 | 🞆 | 🞆 |
| homosexuality? | 🞆 | 🞆 | 🞆 | 🞆 | 🞆 | 🞆 | 🞆 | 🞆 |
| conservative ideas/values? | 🞆 | 🞆 | 🞆 | 🞆 | 🞆 | 🞆 | 🞆 | 🞆 |
| liberal ideas/values? | 🞆 | 🞆 | 🞆 | 🞆 | 🞆 | 🞆 | 🞆 | 🞆 |

**Respect Measurement for Islam, Homosexuality, Conservatives and Liberals**

**Direct respect measurement.**

How much do you **respect** the following people?

|  | not at all  0 | slightly  1 | moderately 2 | much 3 | very much  4 | I cannot  or do not  want to  answer |
| --- | --- | --- | --- | --- | --- | --- |
| Muslims in the U.S.? | 🞆 | 🞆 | 🞆 | 🞆 | 🞆 | 🞆 |
| homosexuals in the U.S.? | 🞆 | 🞆 | 🞆 | 🞆 | 🞆 | 🞆 |
| conservatives in the U.S.? | 🞆 | 🞆 | 🞆 | 🞆 | 🞆 | 🞆 |
| liberals in the U.S.? | 🞆 | 🞆 | 🞆 | 🞆 | 🞆 | 🞆 |

**Equality recognition measurement.**

Do you view the following people as **equal fellow citizens**?

|  | not at all 0 | 1 | 2 | 3 | absolutely 4 | I cannot  or do not  want to  answer |
| --- | --- | --- | --- | --- | --- | --- |
| Muslims in the U.S.? | 🞆 | 🞆 | 🞆 | 🞆 | 🞆 | 🞆 |
| homosexuals in the U.S.? | 🞆 | 🞆 | 🞆 | 🞆 | 🞆 | 🞆 |
| conservatives in the U.S.? | 🞆 | 🞆 | 🞆 | 🞆 | 🞆 | 🞆 |
| liberals in the U.S.? | 🞆 | 🞆 | 🞆 | 🞆 | 🞆 | 🞆 |

**Tolerance Measurement**

Should the following people **be allowed to live the way they want to live**?

|  | not at all -2 | -1 | 0 | +1 | absolutely +2 | I cannot  or do not  want to  answer |
| --- | --- | --- | --- | --- | --- | --- |
| Muslims in the U.S.? | 🞆 | 🞆 | 🞆 | 🞆 | 🞆 | 🞆 |
| homosexuals in the U.S.? | 🞆 | 🞆 | 🞆 | 🞆 | 🞆 | 🞆 |
| conservatives in the U.S.? | 🞆 | 🞆 | 🞆 | 🞆 | 🞆 | 🞆 |
| liberals in the U.S.? | 🞆 | 🞆 | 🞆 | 🞆 | 🞆 | 🞆 |

**Alevis in Germany (Study 5)**

Participants could choose the language of the survey to be either German or Turkish. Both versions are presented below. The following items were measured at all time points:

**Approval or Disapproval of Christians, Jews, Sunnis, Atheists, Homosexuality and Feminism**

|  | sehr schlecht -3 | ziemlich schlecht -2 | etwas schlecht -1 | neutral 0 | etwas gut +1 | ziemlich gut +2 | sehr gut +3 | kann oder will ich nicht beantworten |
| --- | --- | --- | --- | --- | --- | --- | --- | --- |
| Ist das Christentum etwas Schlechtes oder etwas Gutes? | 🞆 | 🞆 | 🞆 | 🞆 | 🞆 | 🞆 | 🞆 | 🞆 |
| Ist das Judentum etwas Schlechtes oder etwas Gutes? | 🞆 | 🞆 | 🞆 | 🞆 | 🞆 | 🞆 | 🞆 | 🞆 |
| Ist das Sunnitentum etwas Schlechtes oder etwas Gutes? | 🞆 | 🞆 | 🞆 | 🞆 | 🞆 | 🞆 | 🞆 | 🞆 |
| Ist ein Leben ohne Glauben an Gott etwas Schlechtes oder etwas Gutes? | 🞆 | 🞆 | 🞆 | 🞆 | 🞆 | 🞆 | 🞆 | 🞆 |
| Ist Homosexualität etwas Schlechtes oder etwas Gutes? | 🞆 | 🞆 | 🞆 | 🞆 | 🞆 | 🞆 | 🞆 | 🞆 |
| Ist Feminismus etwas Schlechtes oder etwas Gutes? | 🞆 | 🞆 | 🞆 | 🞆 | 🞆 | 🞆 | 🞆 | 🞆 |

|  | çok kötü  -3 | oldukça kötü  -2 | biraz kötü -1 | tarafsız  0 | biraz iyi +1 | oldukça iyi +2 | çok  iyi +3 | yanıtlayamı-  yorum veya yanıtlamak istemiyorum |
| --- | --- | --- | --- | --- | --- | --- | --- | --- |
| **Hıristiyanlık** kötü bir şey mi yoksa iyi bir şey midir? | 🞆 | 🞆 | 🞆 | 🞆 | 🞆 | 🞆 | 🞆 | 🞆 |
| **Musevîlik** kötü bir şey mi yoksa iyi bir şey midir? | 🞆 | 🞆 | 🞆 | 🞆 | 🞆 | 🞆 | 🞆 | 🞆 |
| **Sünnîlik** kötü bir şey mi yoksa iyi bir şey midir? | 🞆 | 🞆 | 🞆 | 🞆 | 🞆 | 🞆 | 🞆 | 🞆 |
| **Tanrıya (Allah’a) inançtan yoksun bir yaşam** kötü bir şey mi yoksa iyi bir şey midir? | 🞆 | 🞆 | 🞆 | 🞆 | 🞆 | 🞆 | 🞆 | 🞆 |
| **Eşcinsellik** kötü bir şey mi yoksa iyi bir şey midir? | 🞆 | 🞆 | 🞆 | 🞆 | 🞆 | 🞆 | 🞆 | 🞆 |
| **Feminizm** kötü bir şey mi yoksa iyi bir şey midir? | 🞆 | 🞆 | 🞆 | 🞆 | 🞆 | 🞆 | 🞆 | 🞆 |

**Respect Measurement for Christians, Jews, Sunnis, Atheists, Homosexuality and Feminism**

**Direct respect measurement.**

Welche Behandlung verdienen Ihrer Meinung nach die folgenden Gruppen?

|  | stimme nicht zu  0 | stimme wenig zu  1 | stimme mittelmäßig zu 2 | stimme ziemlich zu 3 | stimme sehr zu  4 | kann oder will ich nicht beantworten |
| --- | --- | --- | --- | --- | --- | --- |
| Christen in Deutschland verdienen Respekt | 🞆 | 🞆 | 🞆 | 🞆 | 🞆 | 🞆 |
| Juden in Deutschland verdienen Respekt | 🞆 | 🞆 | 🞆 | 🞆 | 🞆 | 🞆 |
| Sunniten in Deutschland verdienen Respekt | 🞆 | 🞆 | 🞆 | 🞆 | 🞆 | 🞆 |
| Menschen ohne Glauben in Deutschland verdienen Respekt | 🞆 | 🞆 | 🞆 | 🞆 | 🞆 | 🞆 |
| Homosexuelle in Deutschland verdienen Respekt | 🞆 | 🞆 | 🞆 | 🞆 | 🞆 | 🞆 |
| Feministinnen in Deutschland verdienen Respekt | 🞆 | 🞆 | 🞆 | 🞆 | 🞆 | 🞆 |

Aşağıdaki gruplar görüşünüze göre hangi davranışı hak ediyorlar?

|  | kesinlikle katılmıyorum 0 | katılmı-yorum 1 | kararsızım 2 | katılıyorum 3 | kesinlikle katılı  yorum 4 | yanıtlayamı-  yorum veya yanıtlamak istemiyorum |
| --- | --- | --- | --- | --- | --- | --- |
| Almanya’daki Hiristiyanlar saygıyı hak ediyorlar | 🞆 | 🞆 | 🞆 | 🞆 | 🞆 | 🞆 |
| Almanya’daki Musevîler saygıyı hak ediyorlar | 🞆 | 🞆 | 🞆 | 🞆 | 🞆 | 🞆 |
| Almanya’daki Sünnîler saygıyı hak ediyorlar | 🞆 | 🞆 | 🞆 | 🞆 | 🞆 | 🞆 |
| Almanya’daki inanç sahibi olmayan insanlar saygıyı hak ediyorlar | 🞆 | 🞆 | 🞆 | 🞆 | 🞆 | 🞆 |
| Almanya’daki eşcinseller saygıyı hak ediyorlar | 🞆 | 🞆 | 🞆 | 🞆 | 🞆 | 🞆 |
| Almanya’daki feminist kadınlar saygıyı hak ediyorlar | 🞆 | 🞆 | 🞆 | 🞆 | 🞆 | 🞆 |

**Equality recognition measurement.**

Welche Behandlung verdienen Ihrer Meinung nach die folgenden Gruppen?

|  | stimme nicht zu  0 | stimme wenig zu  1 | stimme mittelmäßig zu 2 | stimme ziemlich zu 3 | stimme sehr zu  4 | kann oder will ich nicht beantworten |
| --- | --- | --- | --- | --- | --- | --- |
| Christen in Deutschland verdienen Behandlung als gleichwertige Mitbürger | 🞆 | 🞆 | 🞆 | 🞆 | 🞆 | 🞆 |
| Juden in Deutschland verdienen Behandlung als gleichwertige Mitbürger | 🞆 | 🞆 | 🞆 | 🞆 | 🞆 | 🞆 |
| Sunniten in Deutschland verdienen Behandlung als gleichwertige Mitbürger | 🞆 | 🞆 | 🞆 | 🞆 | 🞆 | 🞆 |
| Menschen ohne Glauben in Deutschland verdienen Behandlung als gleichwertige Mitbürger | 🞆 | 🞆 | 🞆 | 🞆 | 🞆 | 🞆 |
| Homosexuelle in Deutschland verdienen Behandlung als gleichwertige Mitbürger | 🞆 | 🞆 | 🞆 | 🞆 | 🞆 | 🞆 |
| Feministinnen in Deutschland verdienen Behandlung als gleichwertige Mitbürger | 🞆 | 🞆 | 🞆 | 🞆 | 🞆 | 🞆 |

Aşağıdaki gruplar görüşünüze göre hangi davranışı hak ediyorlar?

|  | kesinlikle katılmıyorum 0 | katılmı-yorum 1 | kararsızım 2 | katılıyorum 3 | kesinlikle katılı  yorum 4 | yanıtlayamı-  yorum veya yanıtlamak istemiyorum |
| --- | --- | --- | --- | --- | --- | --- |
| Almanya’daki Hiristiyanlar kendilerine eşit vatandaşlar olarak davranılmasını hak ediyorlar | 🞆 | 🞆 | 🞆 | 🞆 | 🞆 | 🞆 |
| Almanya’daki Musevîler kendilerine eşit vatandaşlar olarak davranılmasını hak ediyorlar | 🞆 | 🞆 | 🞆 | 🞆 | 🞆 | 🞆 |
| Almanya’daki Sünnîler kendilerine eşit vatandaşlar olarak davranılmasını hak ediyorlar | 🞆 | 🞆 | 🞆 | 🞆 | 🞆 | 🞆 |
| Almanya’daki inanç sahibi olmayan insanlar kendilerine eşit vatandaşlar olarak davranılmasını hak ediyorlar | 🞆 | 🞆 | 🞆 | 🞆 | 🞆 | 🞆 |
| Almanya’daki eşcinseller kendilerine eşit vatandaşlar olarak davranılmasını hak ediyorlar | 🞆 | 🞆 | 🞆 | 🞆 | 🞆 | 🞆 |
| Almanya’daki feminist kadınlar kendilerine eşit vatandaşlar olarak davranılmasını hak ediyorlar | 🞆 | 🞆 | 🞆 | 🞆 | 🞆 | 🞆 |

**Tolerance Measurement**

Sollen folgende Gruppen in Deutschland so leben dürfen, wie die jeweilige Gruppe es möchte?

|  | nein, keinesfalls -2 | -1 | 0 | +1 | ja,  unbedingt +2 | kann oder will ich nicht beantworten |
| --- | --- | --- | --- | --- | --- | --- |
| Chrsiten in Deutschland | 🞆 | 🞆 | 🞆 | 🞆 | 🞆 | 🞆 |
| Juden in Deutschland | 🞆 | 🞆 | 🞆 | 🞆 | 🞆 | 🞆 |
| Sunniten in Deutschland | 🞆 | 🞆 | 🞆 | 🞆 | 🞆 | 🞆 |
| Menschen ohne Glauben in Deutschland | 🞆 | 🞆 | 🞆 | 🞆 | 🞆 | 🞆 |
| Homosexuelle in Deutschland | 🞆 | 🞆 | 🞆 | 🞆 | 🞆 | 🞆 |
| Feministinnen in Deutschland | 🞆 | 🞆 | 🞆 | 🞆 | 🞆 | 🞆 |

Aşağıdaki gruplar Almanya’da kendi diledikleri gibi yaşayabilsinler mi?

|  | hayır,  asla -2 | -1 | 0 | +1 | evet, mutlaka +2 | yanıtlayamı-yorum veya yanıtlamak istemiyorum |
| --- | --- | --- | --- | --- | --- | --- |
| Almanya’daki Hıristiyanlar | 🞆 | 🞆 | 🞆 | 🞆 | 🞆 | 🞆 |
| Almanya’daki Musevîler | 🞆 | 🞆 | 🞆 | 🞆 | 🞆 | 🞆 |
| Almanya’daki Sünnîler | 🞆 | 🞆 | 🞆 | 🞆 | 🞆 | 🞆 |
| Almanya’daki inanç sahibi olmayan insanlar | 🞆 | 🞆 | 🞆 | 🞆 | 🞆 | 🞆 |
| Almanya’daki eşcinseller | 🞆 | 🞆 | 🞆 | 🞆 | 🞆 | 🞆 |
| Almanya’daki feminist kadınlar | 🞆 | 🞆 | 🞆 | 🞆 | 🞆 | 🞆 |

**Catholics in Poland (Study 6)**

The following items were measured at all time points:

**Approval or Disapproval of Orthodox, Islam, Atheists, Homosexuality and Feminism**

Prosimy o odpowiedzenie na poniższe pytania, wybierając jedną z możliwych odpowiedzi. Proszę wybrać tą, która jest najbardziej prawdopodobna.

|  | bardzo źle -3 | nie najlepiej -2 | trochę źle -1 | neutralnie  0 | trochę dobrze +1 | dość dobrze +2 | bardzo dobrze +3 | nie chcę lub nie potrafię odpowiedzieć |
| --- | --- | --- | --- | --- | --- | --- | --- | --- |
| Czy wiara prawosławna jest czymś złym czy czymś dobrym? | 🞆 | 🞆 | 🞆 | 🞆 | 🞆 | 🞆 | 🞆 | 🞆 |
| Czy wiara muzułmańska jest czymś złym czy czymś dobrym? | 🞆 | 🞆 | 🞆 | 🞆 | 🞆 | 🞆 | 🞆 | 🞆 |
| Czy życie bez wiary w Boga jest czymś złym czy czymś dobrym? | 🞆 | 🞆 | 🞆 | 🞆 | 🞆 | 🞆 | 🞆 | 🞆 |
| Czy homoseksualizm jest czymś złym czy czymś dobrym? | 🞆 | 🞆 | 🞆 | 🞆 | 🞆 | 🞆 | 🞆 | 🞆 |
| Czy feminizm jest czymś złym czy czymś dobrym? | 🞆 | 🞆 | 🞆 | 🞆 | 🞆 | 🞆 | 🞆 | 🞆 |
| Czy wiara prawosławna jest czymś złym czy czymś dobrym? | 🞆 | 🞆 | 🞆 | 🞆 | 🞆 | 🞆 | 🞆 | 🞆 |

**Respect Measurement for Orthodox, Islam, Atheists, Homosexuality and Feminism**

**Direct respect measurement.**

W jakim stopniu respektuje Pani/Pan następujące osoby?

|  | wcale 0 | trochę  1 | średnio 2 | dosyć 3 | całkowicie  4 | nie chcę lub nie potrafię odpowiedzieć |
| --- | --- | --- | --- | --- | --- | --- |
| prawosławni w Polsce | 🞆 | 🞆 | 🞆 | 🞆 | 🞆 | 🞆 |
| muzułmanie w Polsce | 🞆 | 🞆 | 🞆 | 🞆 | 🞆 | 🞆 |
| niewierzący w Polsce | 🞆 | 🞆 | 🞆 | 🞆 | 🞆 | 🞆 |
| homoseksualiści w Polsce | 🞆 | 🞆 | 🞆 | 🞆 | 🞆 | 🞆 |
| feministki w Polsce | 🞆 | 🞆 | 🞆 | 🞆 | 🞆 | 🞆 |
| prawosławni w Polsce | 🞆 | 🞆 | 🞆 | 🞆 | 🞆 | 🞆 |

**Equality recognition measurement.**

W jakim stopniu uznaje Pani/Pan członków poniższych grup jako równowartościowych obywateli?

|  | wcale 0 | trochę  1 | średnio 2 | dosyć 3 | całkowicie  4 | nie chcę lub nie potrafię odpowiedzieć |
| --- | --- | --- | --- | --- | --- | --- |
| prawosławni w Polsce | 🞆 | 🞆 | 🞆 | 🞆 | 🞆 | 🞆 |
| muzułmanie w Polsce | 🞆 | 🞆 | 🞆 | 🞆 | 🞆 | 🞆 |
| niewierzący w Polsce | 🞆 | 🞆 | 🞆 | 🞆 | 🞆 | 🞆 |
| homoseksualiści w Polsce | 🞆 | 🞆 | 🞆 | 🞆 | 🞆 | 🞆 |
| feministki w Polsce | 🞆 | 🞆 | 🞆 | 🞆 | 🞆 | 🞆 |
| prawosławni w Polsce | 🞆 | 🞆 | 🞆 | 🞆 | 🞆 | 🞆 |

**Tolerance Measurement**

Czy poniższe grupy w Polsce powinny żyć według własnego upodobania?

|  | nie, w żadnym razie -2 | -1 | 0 | +1 | tak, koniecznie +2 | nie chcę lub nie potrafię odpowiedzieć |
| --- | --- | --- | --- | --- | --- | --- |
| prawosławni w Polsce | 🞆 | 🞆 | 🞆 | 🞆 | 🞆 | 🞆 |
| muzułmanie w Polsce | 🞆 | 🞆 | 🞆 | 🞆 | 🞆 | 🞆 |
| niewierzący w Polsce | 🞆 | 🞆 | 🞆 | 🞆 | 🞆 | 🞆 |
| homoseksualiści w Polsce | 🞆 | 🞆 | 🞆 | 🞆 | 🞆 | 🞆 |
| feministki w Polsce | 🞆 | 🞆 | 🞆 | 🞆 | 🞆 | 🞆 |
| prawosławni w Polsce | 🞆 | 🞆 | 🞆 | 🞆 | 🞆 | 🞆 |

**LGBT in the US (Study 7)**

The following items were measured at all time points:

**Approval or Disapproval of Latinos/Hispanics, Asian Americans, African Americans, members of the Tea Party movement, and religious people**

Do you regard the following beliefs and practices as something **good** or something **bad**?

|  | clearly bad -3 | somewhat bad -2 | slightly bad -1 | neutral 0 | slightly good +1 | somewhat good +2 | clearly good +3 | I cannot  or do not  want to  answer |
| --- | --- | --- | --- | --- | --- | --- | --- | --- |
| Latinos/Hispanics | 🞆 | 🞆 | 🞆 | 🞆 | 🞆 | 🞆 | 🞆 | 🞆 |
| Asian Americans^1^ | 🞆 | 🞆 | 🞆 | 🞆 | 🞆 | 🞆 | 🞆 | 🞆 |
| African Americans | 🞆 | 🞆 | 🞆 | 🞆 | 🞆 | 🞆 | 🞆 | 🞆 |
| members of the Tea Party movement | 🞆 | 🞆 | 🞆 | 🞆 | 🞆 | 🞆 | 🞆 | 🞆 |
| religious people | 🞆 | 🞆 | 🞆 | 🞆 | 🞆 | 🞆 | 🞆 | 🞆 |

**Respect Measurement for Latinos/Hispanics, Asian Americans, African Americans, members of the Tea Party movement, and religious people**

**Direct respect measurement.**

How much do you **respect** the following people?

|  | not at all  0 | slightly  1 | moderately 2 | much 3 | very much  4 | I cannot  or do not  want to  answer |
| --- | --- | --- | --- | --- | --- | --- |
| Latinos/Hispanics | 🞆 | 🞆 | 🞆 | 🞆 | 🞆 | 🞆 |
| Asian Americans | 🞆 | 🞆 | 🞆 | 🞆 | 🞆 | 🞆 |
| African Americans | 🞆 | 🞆 | 🞆 | 🞆 | 🞆 | 🞆 |
| members of the Tea Party movement | 🞆 | 🞆 | 🞆 | 🞆 | 🞆 | 🞆 |
| religious people | 🞆 | 🞆 | 🞆 | 🞆 | 🞆 | 🞆 |

**Equality recognition measurement.**

Do you view the following people as **equal fellow citizens**?

|  | not at all 0 | 1 | 2 | 3 | absolutely 4 | I cannot  or do not  want to  answer |
| --- | --- | --- | --- | --- | --- | --- |
| Latinos/Hispanics | 🞆 | 🞆 | 🞆 | 🞆 | 🞆 | 🞆 |
| Asian Americans | 🞆 | 🞆 | 🞆 | 🞆 | 🞆 | 🞆 |
| African Americans | 🞆 | 🞆 | 🞆 | 🞆 | 🞆 | 🞆 |
| members of the Tea Party movement | 🞆 | 🞆 | 🞆 | 🞆 | 🞆 | 🞆 |
| religious people | 🞆 | 🞆 | 🞆 | 🞆 | 🞆 | 🞆 |

**Tolerance Measurement**

Should the following people **be allowed to live the way they want to live**?

|  | not at all -2 | -1 | 0 | +1 | absolutely +2 | I cannot  or do not  want to  answer |
| --- | --- | --- | --- | --- | --- | --- |
| Latinos/Hispanics | 🞆 | 🞆 | 🞆 | 🞆 | 🞆 | 🞆 |
| Asian Americans | 🞆 | 🞆 | 🞆 | 🞆 | 🞆 | 🞆 |
| African Americans | 🞆 | 🞆 | 🞆 | 🞆 | 🞆 | 🞆 |
| members of the Tea Party movement | 🞆 | 🞆 | 🞆 | 🞆 | 🞆 | 🞆 |
| religious people | 🞆 | 🞆 | 🞆 | 🞆 | 🞆 | 🞆 |

**LGBT in Germany (Study 8)**

The following items were measured at all time points:

**Approval or Disapproval of Catholics, Protestants, Muslims, Refugees, Feminism and Members of the Political Party Alternative for Germany (AfD)**

|  | sehr schlecht -3 | ziemlich schlecht -2 | etwas schlecht -1 | neutral 0 | etwas gut +1 | ziemlich gut +2 | sehr gut +3 | kann oder will ich nicht beantworten |
| --- | --- | --- | --- | --- | --- | --- | --- | --- |
| Ist das katholische Christentum etwas Schlechtes oder etwas Gutes? | 🞆 | 🞆 | 🞆 | 🞆 | 🞆 | 🞆 | 🞆 | 🞆 |
| Ist das evangelische Christentum etwas Schlechtes oder etwas Gutes? | 🞆 | 🞆 | 🞆 | 🞆 | 🞆 | 🞆 | 🞆 | 🞆 |
| Ist der Islam etwas Schlechtes oder etwas Gutes? | 🞆 | 🞆 | 🞆 | 🞆 | 🞆 | 🞆 | 🞆 | 🞆 |
| Ist die Aufnahme von Flüchtlingen in Deutschland etwas Schlechtes oder etwas Gutes? | 🞆 | 🞆 | 🞆 | 🞆 | 🞆 | 🞆 | 🞆 | 🞆 |
| Ist Feminismus etwas Schlechtes oder etwas Gutes? | 🞆 | 🞆 | 🞆 | 🞆 | 🞆 | 🞆 | 🞆 | 🞆 |
| Sind die Ziele der AfD etwas Schlechtes oder etwas Gutes? | 🞆 | 🞆 | 🞆 | 🞆 | 🞆 | 🞆 | 🞆 | 🞆 |

**Respect Measurement for Catholics, Protestants, Muslims, Refugees, Feminists, and Members of the Political Party Alternative for Germany (AfD)**

**Direct respect measurement.**

Ich respektiere die folgenden Personen.

|  | gar nicht 0 | wenig 1 | mittelmäßig 2 | ziemlich 3 | völlig  4 | kann oder will ich nicht beantworten |
| --- | --- | --- | --- | --- | --- | --- |
| katholische Christ*innen in Deutschland | 🞆 | 🞆 | 🞆 | 🞆 | 🞆 | 🞆 |
| evangelische Christ*innen in Deutschland | 🞆 | 🞆 | 🞆 | 🞆 | 🞆 | 🞆 |
| Muslim*innen in Deutschland | 🞆 | 🞆 | 🞆 | 🞆 | 🞆 | 🞆 |
| Flüchtlinge in Deutschland | 🞆 | 🞆 | 🞆 | 🞆 | 🞆 | 🞆 |
| Feminist*innen in Deutschland | 🞆 | 🞆 | 🞆 | 🞆 | 🞆 | 🞆 |
| Anhänger*innen der AfD | 🞆 | 🞆 | 🞆 | 🞆 | 🞆 | 🞆 |

**Equality recognition measurement.**

Ich betrachte die folgenden Personen als gleichwertige Mitbürger*innen.

|  | gar nicht 0 | wenig 1 | mittelmäßig 2 | ziemlich 3 | völlig  4 | kann oder will ich nicht beantworten |
| --- | --- | --- | --- | --- | --- | --- |
| katholische Christ*innen in Deutschland | 🞆 | 🞆 | 🞆 | 🞆 | 🞆 | 🞆 |
| evangelische Christ*innen in Deutschland | 🞆 | 🞆 | 🞆 | 🞆 | 🞆 | 🞆 |
| Muslim*innen in Deutschland | 🞆 | 🞆 | 🞆 | 🞆 | 🞆 | 🞆 |
| Flüchtlinge in Deutschland | 🞆 | 🞆 | 🞆 | 🞆 | 🞆 | 🞆 |
| Feminist*innen in Deutschland | 🞆 | 🞆 | 🞆 | 🞆 | 🞆 | 🞆 |
| Anhänger*innen der AfD | 🞆 | 🞆 | 🞆 | 🞆 | 🞆 | 🞆 |

**Tolerance Measurement**

Sollen die folgenden Gruppen in Deutschland so leben dürfen, wie deren Mitglieder es möchten?

|  | nein, keinesfalls -2 | -1 | 0 | +1 | ja, unbedingt +2 | kann oder will ich nicht beantworten |
| --- | --- | --- | --- | --- | --- | --- |
| katholische Christ*innen in Deutschland | 🞆 | 🞆 | 🞆 | 🞆 | 🞆 | 🞆 |
| evangelische Christ*innen in Deutschland | 🞆 | 🞆 | 🞆 | 🞆 | 🞆 | 🞆 |
| Muslim*innen in Deutschland | 🞆 | 🞆 | 🞆 | 🞆 | 🞆 | 🞆 |
| Flüchtlinge in Deutschland | 🞆 | 🞆 | 🞆 | 🞆 | 🞆 | 🞆 |
| Feminist*innen in Deutschland | 🞆 | 🞆 | 🞆 | 🞆 | 🞆 | 🞆 |
| Anhänger*innen der AfD | 🞆 | 🞆 | 🞆 | 🞆 | 🞆 | 🞆 |

**Catholics in Poland (Study 9)**

The following items were used in a cross-sectional survey:

**Approval or Disapproval of Orthodox, Islam, Atheists, Homosexuality and Feminism**

Prosimy o odpowiedzenie na poniższe pytania, wybierając jedną z możliwych odpowiedzi. Proszę wybrać tą, która jest najbardziej prawdopodobna.

|  | bardzo źle -3 | nie najlepiej -2 | trochę źle -1 | neutralnie  0 | trochę dobrze +1 | dość dobrze +2 | bardzo dobrze +3 | nie chcę lub nie potrafię odpowiedzieć |
| --- | --- | --- | --- | --- | --- | --- | --- | --- |
| Czy wiara prawosławna jest czymś złym czy czymś dobrym? | 🞆 | 🞆 | 🞆 | 🞆 | 🞆 | 🞆 | 🞆 | 🞆 |
| Czy wiara muzułmańska jest czymś złym czy czymś dobrym? | 🞆 | 🞆 | 🞆 | 🞆 | 🞆 | 🞆 | 🞆 | 🞆 |
| Czy życie bez wiary w Boga jest czymś złym czy czymś dobrym? | 🞆 | 🞆 | 🞆 | 🞆 | 🞆 | 🞆 | 🞆 | 🞆 |
| Czy homoseksualizm jest czymś złym czy czymś dobrym? | 🞆 | 🞆 | 🞆 | 🞆 | 🞆 | 🞆 | 🞆 | 🞆 |
| Czy feminizm jest czymś złym czy czymś dobrym? | 🞆 | 🞆 | 🞆 | 🞆 | 🞆 | 🞆 | 🞆 | 🞆 |
| Czy wiara prawosławna jest czymś złym czy czymś dobrym? | 🞆 | 🞆 | 🞆 | 🞆 | 🞆 | 🞆 | 🞆 | 🞆 |

**Respect Measurement for Orthodox, Islam, Atheists, Homosexuality and Feminism**

**Direct respect measurement.**

Respektuję następujące osoby.

|  | wcale 0 | trochę  1 | średnio 2 | dosyć 3 | całkowicie  4 | nie chcę lub nie potrafię odpowiedzieć |
| --- | --- | --- | --- | --- | --- | --- |
| prawosławni w Polsce | 🞆 | 🞆 | 🞆 | 🞆 | 🞆 | 🞆 |
| muzułmanie w Polsce | 🞆 | 🞆 | 🞆 | 🞆 | 🞆 | 🞆 |
| niewierzący w Polsce | 🞆 | 🞆 | 🞆 | 🞆 | 🞆 | 🞆 |
| homoseksualiści w Polsce | 🞆 | 🞆 | 🞆 | 🞆 | 🞆 | 🞆 |
| feministki w Polsce | 🞆 | 🞆 | 🞆 | 🞆 | 🞆 | 🞆 |
| prawosławni w Polsce | 🞆 | 🞆 | 🞆 | 🞆 | 🞆 | 🞆 |

**Equality recognition measurement.**

Uznaję członków poniższych grup jako równowartościowych obywateli.

|  | wcale 0 | trochę  1 | średnio 2 | dosyć 3 | całkowicie  4 | nie chcę lub nie potrafię odpowiedzieć |
| --- | --- | --- | --- | --- | --- | --- |
| prawosławni w Polsce | 🞆 | 🞆 | 🞆 | 🞆 | 🞆 | 🞆 |
| muzułmanie w Polsce | 🞆 | 🞆 | 🞆 | 🞆 | 🞆 | 🞆 |
| niewierzący w Polsce | 🞆 | 🞆 | 🞆 | 🞆 | 🞆 | 🞆 |
| homoseksualiści w Polsce | 🞆 | 🞆 | 🞆 | 🞆 | 🞆 | 🞆 |
| feministki w Polsce | 🞆 | 🞆 | 🞆 | 🞆 | 🞆 | 🞆 |
| prawosławni w Polsce | 🞆 | 🞆 | 🞆 | 🞆 | 🞆 | 🞆 |

**Tolerance Measurement**

Czy poniższe grupy w Polsce powinny żyć według własnego upodobania?

|  | nie, w żadnym razie -2 | -1 | 0 | +1 | tak, koniecznie +2 | nie chcę lub nie potrafię odpowiedzieć |
| --- | --- | --- | --- | --- | --- | --- |
| prawosławni w Polsce | 🞆 | 🞆 | 🞆 | 🞆 | 🞆 | 🞆 |
| muzułmanie w Polsce | 🞆 | 🞆 | 🞆 | 🞆 | 🞆 | 🞆 |
| niewierzący w Polsce | 🞆 | 🞆 | 🞆 | 🞆 | 🞆 | 🞆 |
| homoseksualiści w Polsce | 🞆 | 🞆 | 🞆 | 🞆 | 🞆 | 🞆 |
| feministki w Polsce | 🞆 | 🞆 | 🞆 | 🞆 | 🞆 | 🞆 |
| prawosławni w Polsce | 🞆 | 🞆 | 🞆 | 🞆 | 🞆 | 🞆 |

**Experiment Catholics in Poland (Study 10)**

**Verbatim Manipulation of High Respect**

Prawdopodobnie polskie społeczeństwo składać się będzie z coraz różnorodniejszych grup społecznych. Jesteśmy zainteresowani jakie spotkania między tymi różnorodnymi grupami mogą mieć miejsca i jak ich uczestnicy je odczuwają.

Proszę dokładnie przypomnieć sobie sytuację w której grupa katolików w Polsce była przez polskie społeczeństwo traktowana i ceniona jako [grupa wobec innych grup równowartościowa.

Jeżeli nie możecie sobie przypomnieć Państwo takiej sytuacji proszę dokładnie wyobrazić sobie taką sytuacje.

Proszę opisać hasłami jaka sytuacja się Państwu przypomniała lub jaką sytuacje Państwo sobie wyobrazili i jak ją odbieracie:

**Verbatim Manipulation of Low Respect**

Prawdopodobnie polskie społeczeństwo składać się będzie z coraz różnorodniejszych grup społecznych. Jesteśmy zainteresowani jakie spotkania między tymi różnorodnymi grupami mogą mieć miejsca i jak ich uczestnicy je odczuwają.

Proszę dokładnie przypomnieć sobie sytuację w której grupa katolików w Polsce była przez polskie społeczeństwo traktowana i ceniona jako grupa wobec innych grup małowartościowa.

Jeżeli nie możecie sobie przypomnieć Państwo takiej sytuacji proszę dokładnie wyobrazić sobie taką sytuacje.

Proszę opisać hasłami jaka sytuacja się Państwu przypomniała lub jaką sytuacje Państwo sobie wyobrazili i jak ją odbieracie:

**Verbatim Assessment of the Manipulation Check**

Przez tą sytuacje czułam/czułem się lub czuła/czuł bym się:

|  | wcale  0 | troche 1 | średnio 2 | dosyć 3 | całkowicie  4 | nie chcę lub nie potrafię odpowiedzieć |
| --- | --- | --- | --- | --- | --- | --- |
| jako równowartościowa/-y obywatelka/obywatel Polski | 🞆 | 🞆 | 🞆 | 🞆 | 🞆 | 🞆 |
| jako małowartościowa/-y obywatelka/obywatel Polski | 🞆 | 🞆 | 🞆 | 🞆 | 🞆 | 🞆 |
| szanowana/szanowany | 🞆 | 🞆 | 🞆 | 🞆 | 🞆 | 🞆 |
| tolerowana/tolerowany | 🞆 | 🞆 | 🞆 | 🞆 | 🞆 | 🞆 |
| zdegradowana/zdegradowany | 🞆 | 🞆 | 🞆 | 🞆 | 🞆 | 🞆 |

**Verbatim Tolerance Measurement**

Czy poniższe grupy w Polsce powinny żyć według własnego upodobania?

|  | nie, w żadnym razie -2 | -1 | 0 | +1 | tak, koniecznie +2 | nie chcę lub nie potrafię odpowiedzieć |
| --- | --- | --- | --- | --- | --- | --- |
| prawosławni w Polsce | 🞆 | 🞆 | 🞆 | 🞆 | 🞆 | 🞆 |
| muzułmanie w Polsce | 🞆 | 🞆 | 🞆 | 🞆 | 🞆 | 🞆 |
| niewierzący w Polsce | 🞆 | 🞆 | 🞆 | 🞆 | 🞆 | 🞆 |
| homoseksualiści w Polsce | 🞆 | 🞆 | 🞆 | 🞆 | 🞆 | 🞆 |
| feministki w Polsce | 🞆 | 🞆 | 🞆 | 🞆 | 🞆 | 🞆 |

**Experiment Neues Zentrum 1 (Study 11)**

**Verbatim Description of the Student Group ‚New Center’**

Aktuell werden an verschiedenen deutschen Hochschulen neue Hochschulgruppen gegründet, die in das Studierendenparlament gewählt werden können. Bitte lesen Sie sich die nachfolgende Beschreibung einer neu gegründeten Gruppe gut durch und überlegen Sie, was Sie von dieser Gruppe halten würden, wenn Sie an Ihrer Hochschule zur Wahl stünde.

An Ihrer Hochschule wurde eine neue Hochschulgruppe gegründet. Diese nennt sich "Neues Zentrum" und steht politisch der AfD nahe.

Das "Neue Zentrum" bekennt sich beispielsweise zur Vermittlung auch religiöser Werte im Bildungssystem und möchte mit anderen Studierenden darüber in den Dialog treten, inwiefern Religiosität einen Platz auf dem Campus haben kann oder haben sollte.

Zudem legt diese Hochschulgruppe Wert auf ein angemessenes Sicherheitskonzept und fordert beispielsweise mehr Sicherheitspersonal für den Campus.

Die Zivilklausel wird vom "Neuen Zentrum" abgelehnt. Wissenschaftler müssten sowohl zu zivilen Zwecken als auch zu nicht-zivilen, also auch zu militärischen Zwecken, forschen dürfen. In Zeiten unterfinanzierter Hochschulen müsste Sponsoring aus der Industrie oder von der Bundeswehr jederzeit willkommen sein.

Das "Neue Zentrum" sieht die fortschreitende Internationalisierung deutscher Universitäten zudem kritisch. Die Gruppe lehnt den Vorschlag ab, bestimmte Master-Studiengänge nur auf Englisch anzubieten, und legt zudem Wert darauf, dass deutsche Studierende an deutschen Universitäten Vorrang vor internationalen Studierenden haben sollen.

**Verbatim Assessment of Disapproval Before the Manipulation**

Was würden Sie sagen: Sind die Ziele dieser Hochschulgruppe etwas Schlechtes oder etwas Gutes?

| etwas Schlechtes -3 | -2 | -1 | 0 | +1 | +2 | etwas  Gutes +3 |
| --- | --- | --- | --- | --- | --- | --- |
| 🞆 | 🞆 | 🞆 | 🞆 | 🞆 | 🞆 | 🞆 |

Wie würden Sie einer solchen Gruppe gegenüberstehen?

|  | stimme gar nicht zu 1 | 2 | 3 | 4 | 5 | 6 | stimme voll zu 7 |
| --- | --- | --- | --- | --- | --- | --- | --- |
| Einer solchen Hochschulgruppe würde ich ablehnend gegenüberstehen. | 🞆 | 🞆 | 🞆 | 🞆 | 🞆 | 🞆 | 🞆 |
| Einer solchen Hochschulgruppe würde ich positiv gegenüberstehen. | 🞆 | 🞆 | 🞆 | 🞆 | 🞆 | 🞆 | 🞆 |

**Verbatim Manipulation of High Respect**

Eine Kurzumfrage unter Studierenden verschiedener Hochschulen hat ergeben, dass 75% aller befragten Studierenden versuchen würden, Mitglieder einer Hochschulgruppe wie dem „Neuen Zentrum“ in der Hochschulpolitik als gleichberechtigte Gesprächspartner zu respektieren. 83% sagten es sei wichtig, auch den Mitgliedern einer solchen Gruppe ein gleichberechtigtes Rederecht einzuräumen, sie ausreden zu lassen und sich mit den vorgebrachten Argumenten auseinanderzusetzen. Etwa zwei Drittel aller Studierenden (69%) sagte außerdem, es gäbe keine guten Gründe das „Neue Zentrum“ anders zu behandeln als andere Hochschulgruppen

**Verbatim Manipulation of Low respect**

Eine Kurzumfrage unter Studierenden verschiedener Hochschulen hat ergeben, dass nur 25% aller befragten Studierenden versuchen würden, Mitglieder einer Hochschulgruppe wie dem „Neuen Zentrum“ in der Hochschulpolitik als gleichberechtigte Gesprächspartner zu respektieren. Nur 13% sagten es sei wichtig, auch den Mitgliedern einer solchen Gruppe ein gleichberechtigtes Rederecht einzuräumen, sie ausreden zu lassen und sich mit den vorgebrachten Argumenten auseinanderzusetzen. Etwa zwei Drittel aller Studierenden (69%) sagte außerdem, es gäbe durchaus gute Gründe, das „Neue Zentrum“ anders zu behandeln als andere Hochschulgruppen.

**Verbatim Assessment of the Manipulation Check**

Wie würden Sie persönlich einer Hochschulgruppe wie dem „Neuen Zentrum“ gegenüberstehen?

|  | stimme gar nicht zu 1 | 2 | 3 | 4 | 5 | 6 | stimme voll zu 7 |
| --- | --- | --- | --- | --- | --- | --- | --- |
| Ich würde die Mitglieder der Hochschulgruppe „Neues Zentrum“ als gleichberechtigte Gesprächspartner betrachten. | 🞆 | 🞆 | 🞆 | 🞆 | 🞆 | 🞆 | 🞆 |
| Ich denke, dass man die Mitglieder dieser Hochschulgruppe mit ihrer Meinung ernst nehmen sollte. | 🞆 | 🞆 | 🞆 | 🞆 | 🞆 | 🞆 | 🞆 |
| Ich denke, dass man sich mit den Argumenten der Gruppe genauso auseinandersetzen sollte, wie mit den Argumenten anderer Gruppen. | 🞆 | 🞆 | 🞆 | 🞆 | 🞆 | 🞆 | 🞆 |

**Verbatim Tolerance Measurement**

Wie würden Sie persönlich einer Hochschulgruppe wie dem „Neuen Zentrum“ gegenüberstehen?

Die Mitglieder des „Neuen Zentrums“…

|  | stimme gar nicht zu 1 | 2 | 3 | 4 | 5 | 6 | stimme voll zu 7 |
| --- | --- | --- | --- | --- | --- | --- | --- |
| ... sollen ihre Hochschulgruppe gründen können. | 🞆 | 🞆 | 🞆 | 🞆 | 🞆 | 🞆 | 🞆 |
| ... sollen bei ihrer Meinung bleiben können, auch wenn andere versuchen sie umzustimmen. | 🞆 | 🞆 | 🞆 | 🞆 | 🞆 | 🞆 | 🞆 |
| ... sollen sich in den Räumen der Uni treffen können, um ihre Hochschulgruppe zu organisieren. | 🞆 | 🞆 | 🞆 | 🞆 | 🞆 | 🞆 | 🞆 |
| ... sollen ihre Ansichten frei und offen äußern können. | 🞆 | 🞆 | 🞆 | 🞆 | 🞆 | 🞆 | 🞆 |
| ... sollen sich auf dem Universitätsgelände versammeln und eine Rede halten können. | 🞆 | 🞆 | 🞆 | 🞆 | 🞆 | 🞆 | 🞆 |
| ... sollen auf dem Universitätsgelände keine Rede halten können, wenn die Mehrheit der Studierenden dagegen ist. | 🞆 | 🞆 | 🞆 | 🞆 | 🞆 | 🞆 | 🞆 |
| ... sollen die Möglichkeit haben ihre Interessen in der Hochschulpolitik zu verfolgen, so wie andere Hochschulgruppen auch. | 🞆 | 🞆 | 🞆 | 🞆 | 🞆 | 🞆 | 🞆 |
| ... sollen nicht dieselbe Möglichkeit haben Hochschulpolitik zu gestalten, wie andere Hochschulgruppen auch. | 🞆 | 🞆 | 🞆 | 🞆 | 🞆 | 🞆 | 🞆 |
| ... sollen von den anderen Hochschulgruppen miteinbezogen werden, um Missstände an der Hochschule zu beseitigen. | 🞆 | 🞆 | 🞆 | 🞆 | 🞆 | 🞆 | 🞆 |
| ... sollen nicht in die gemeinsame Arbeit der anderen Hochschulgruppen miteinbezogen werden. | 🞆 | 🞆 | 🞆 | 🞆 | 🞆 | 🞆 | 🞆 |

**Experiment Neues Zentrum 2 (Study 12)**

**Verbatim Manipulation of Low Disapproval**

Aktuell werden an verschiedenen deutschen Hochschulen neue Hochschulgruppen gegründet, die in das Studierendenparlament gewählt werden können. Bitte lesen Sie sich die nachfolgende Beschreibung einer neu gegründeten Gruppe gut durch und überlegen Sie, was Sie von dieser Gruppe halten würden, wenn Sie an Ihrer Hochschule zur Wahl stünde.

An Ihrer Hochschule wurde eine neue Hochschulgruppe gegründet. Diese nennt sich "Neues Zentrum" und steht politisch der CDU nahe.

Das "Neue Zentrum" bekennt sich beispielsweise zur Vermittlung auch religiöser Werte im Bildungssystem und möchte mit anderen Studierenden darüber in Dialog treten, inwiefern Religiösität einen Platz auf dem Campus haben kann oder haben sollte.

Zudem legt diese Hochschulgruppe Wert auf ein angemessenes Sicherheitskonzept und fordert beispielsweise mehr Sicherheitspersonal für den Campus.

Die Zivilklausel wird vom "Neuen Zentrum" abgelehnt. Wissenschaftler müssten sowohl zu zivilen Zwecken als auch zu nicht-zivilen, also auch zu militärischen Zwecken, forschen dürfen. In Zeiten unterfinanzierter Hochschulen müsste Sponsoring aus der Industrie oder von der Bundeswehr jederzeit willkommen sein.

Das "Neue Zentrum" sieht die fortschreitende Internationalisierung deutscher Universitäten zudem positiv. Die Gruppe unterstützt den Vorschlag, bestimmte Master-Studiengänge auf Englisch anzubieten, und legt zudem Wert darauf, dass sich sowohl deutsche Studierende als auch internationale Studierende an deutschen Universitäten wohl fühlen sollen.

**Verbatim Manipulation of High Disapproval**

Aktuell werden an verschiedenen deutschen Hochschulen neue Hochschulgruppen

gegründet, die in das Studierendenparlament gewählt werden können. Bitte lesen

Sie sich die nachfolgende Beschreibung einer neu gegründeten Gruppe gut durch

und überlegen Sie, was Sie von dieser Gruppe halten würden, wenn Sie an Ihrer

Hochschule zur Wahl stünde.

An Ihrer Hochschule wurde eine neue Hochschulgruppe gegründet. Diese nennt sich "Neues Zentrum" und steht politisch zwischen CDU und AFD.

Das "Neue Zentrum" bekennt sich beispielsweise zur Vermittlung auch religiöser Werte im Bildungssystem und möchte mit anderen Studierenden darüber in den Dialog treten, inwiefern Religiösität einen Platz auf dem Campus haben kann oder haben sollte.

Zudem legt diese Hochschulgruppe Wert auf ein angemessenes Sicherheitskonzept und fordert beispielsweise mehr Sicherheitspersonal für den Campus.

Die Zivilklausel wird vom "Neuen Zentrum" abgelehnt. Wissenschaftler müssten sowohl zu zivilen Zwecken als auch zu nicht-zivilen, also auch zu militärischen Zwecken, forschen dürfen. In Zeiten unterfinanzierter Hochschulen müsste Sponsoring aus der Industrie oder von der Bundeswehr jederzeit willkommen sein.

Das "Neue Zentrum" sieht die fortschreitende Internationalisierung deutscher Universitäten zudem kritisch. Die Gruppe lehnt den Vorschlag ab, bestimmte Master-Studiengänge nur auf Englisch anzubieten, und legt zudem Wert darauf, dass deutsche Studierende an deutschen Universitäten Vorrang vor internationalen Studierenden haben sollen.

**Verbatim of the Disapproval Manipulation Check**

Was würden Sie sagen: Sind die Ziele dieser Hochschulgruppe etwas Schlechtes oder etwas Gutes?

| etwas Schlechtes -3 | -2 | -1 | 0 | +1 | +2 | etwas  Gutes +3 |
| --- | --- | --- | --- | --- | --- | --- |
| 🞆 | 🞆 | 🞆 | 🞆 | 🞆 | 🞆 | 🞆 |

Wie würden Sie einer solchen Gruppe gegenüberstehen?

|  | stimme gar nicht zu 1 | 2 | 3 | 4 | 5 | 6 | stimme voll zu 7 |
| --- | --- | --- | --- | --- | --- | --- | --- |
| Einer solchen Hochschulgruppe würde ich ablehnend gegenüberstehen. | 🞆 | 🞆 | 🞆 | 🞆 | 🞆 | 🞆 | 🞆 |
| Einer solchen Hochschulgruppe würde ich positiv gegenüberstehen. | 🞆 | 🞆 | 🞆 | 🞆 | 🞆 | 🞆 | 🞆 |

**Verbatim Manipulation of High Respect**

Eine Kurzumfrage unter Studierenden verschiedener Hochschulen hat ergeben, dass 75% aller befragten Studierenden versuchen würden, Mitglieder einer Hochschulgruppe wie dem „Neuen Zentrum“ in der Hochschulpolitik als gleichberechtigte Gesprächspartner zu respektieren. 83% sagten es sei wichtig, auch den Mitgliedern einer solchen Gruppe ein gleichberechtigtes Rederecht einzuräumen, sie ausreden zu lassen und sich mit den vorgebrachten Argumenten auseinanderzusetzen. Etwa zwei Drittel aller Studierenden (69%) sagte außerdem, es gäbe keine guten Gründe das „Neue Zentrum“ anders zu behandeln als andere Hochschulgruppen.

**Verbatim Manipulation of Low Respect**

Eine Kurzumfrage unter Studierenden verschiedener Hochschulen hat ergeben, dass nur 25% aller befragten Studierenden versuchen würden, Mitglieder einer Hochschulgruppe wie dem „Neuen Zentrum“ in der Hochschulpolitik als gleichberechtigte Gesprächspartner zu respektieren. Nur 13% sagten es sei wichtig, auch den Mitgliedern einer solchen Gruppe ein gleichberechtigtes Rederecht einzuräumen, sie ausreden zu lassen und sich mit den vorgebrachten Argumenten auseinanderzusetzen. Etwa zwei Drittel aller Studierenden (69%) sagte außerdem, es gäbe durchaus gute Gründe das „Neue Zentrum“ anders zu behandeln als andere Hochschulgruppen.

**Verbatim Assessment of the Respect Manipulation Check**

Wie würden Sie persönlich einer Hochschulgruppe wie dem „Neuen Zentrum“ gegenüberstehen?

|  | stimme gar nicht zu 1 | 2 | 3 | 4 | 5 | 6 | stimme voll zu 7 |
| --- | --- | --- | --- | --- | --- | --- | --- |
| Ich würde die Mitglieder der Hochschulgruppe „Neues Zentrum“ als gleichberechtigte Gesprächspartner betrachten. | 🞆 | 🞆 | 🞆 | 🞆 | 🞆 | 🞆 | 🞆 |
| Ich denke, dass man die Mitglieder dieser Hochschulgruppe mit ihrer Meinung ernst nehmen sollte. | 🞆 | 🞆 | 🞆 | 🞆 | 🞆 | 🞆 | 🞆 |
| Ich denke, dass man sich mit den Argumenten der Gruppe genauso auseinandersetzen sollte, wie mit den Argumenten anderer Gruppen. | 🞆 | 🞆 | 🞆 | 🞆 | 🞆 | 🞆 | 🞆 |

**Verbatim Tolerance Measurement**

Wie würden Sie persönlich einer Hochschulgruppe wie dem „Neuen Zentrum“ gegenüberstehen?

Die Mitglieder des „Neuen Zentrums“…

|  | stimme gar nicht zu 1 | 2 | 3 | 4 | 5 | 6 | stimme voll zu 7 |
| --- | --- | --- | --- | --- | --- | --- | --- |
| ... sollen ihre Hochschulgruppe gründen können. | 🞆 | 🞆 | 🞆 | 🞆 | 🞆 | 🞆 | 🞆 |
| ... sollen bei ihrer Meinung bleiben können, auch wenn andere versuchen sie umzustimmen. | 🞆 | 🞆 | 🞆 | 🞆 | 🞆 | 🞆 | 🞆 |
| ... sollen sich in den Räumen der Uni treffen können, um ihre Hochschulgruppe zu organisieren. | 🞆 | 🞆 | 🞆 | 🞆 | 🞆 | 🞆 | 🞆 |
| ... sollen ihre Ansichten frei und offen äußern können. | 🞆 | 🞆 | 🞆 | 🞆 | 🞆 | 🞆 | 🞆 |
| ... sollen sich auf dem Universitätsgelände versammeln und eine Rede halten können. | 🞆 | 🞆 | 🞆 | 🞆 | 🞆 | 🞆 | 🞆 |
| ... sollen auf dem Universitätsgelände keine Rede halten können, wenn die Mehrheit der Studierenden dagegen ist. | 🞆 | 🞆 | 🞆 | 🞆 | 🞆 | 🞆 | 🞆 |
| ... sollen die Möglichkeit haben ihre Interessen in der Hochschulpolitik zu verfolgen, so wie andere Hochschulgruppen auch. | 🞆 | 🞆 | 🞆 | 🞆 | 🞆 | 🞆 | 🞆 |
| ... sollen nicht dieselbe Möglichkeit haben Hochschulpolitik zu gestalten, wie andere Hochschulgruppen auch. | 🞆 | 🞆 | 🞆 | 🞆 | 🞆 | 🞆 | 🞆 |
| ... sollen von den anderen Hochschulgruppen miteinbezogen werden, um Missstände an der Hochschule zu beseitigen. | 🞆 | 🞆 | 🞆 | 🞆 | 🞆 | 🞆 | 🞆 |
| ... sollen nicht in die gemeinsame Arbeit der anderen Hochschulgruppen miteinbezogen werden. | 🞆 | 🞆 | 🞆 | 🞆 | 🞆 | 🞆 | 🞆 |

**Footnote**

^1^ Approval or Disapproval of Asian Americans were only measured at time point 1. Therefore, this target group was excluded from analysis.
